# Mobilome impacts on physiology in the widely used non-toxic mutant *Microcystis aeruginosa* PCC 7806 Δ*mcyB* and toxic wildtype

**DOI:** 10.1186/s12864-024-10839-5

**Published:** 2024-10-03

**Authors:** Gwendolyn F. Stark, Alexander R. Truchon, Steven W. Wilhelm

**Affiliations:** Department of Microbiology, University of Tennessee Knoxville, Knoxville, TN USA

**Keywords:** Transposable elements, *Microcystis*, Genomic rearrangement, Sulfur uptake

## Abstract

The *Microcystis* mobilome is a well-known but understudied component of this bloom-forming cyanobacterium. Through genomic and transcriptomic comparisons, we found five families of transposases that altered the expression of genes in the well-studied toxigenic type-strain, *Microcystis aeruginosa* PCC 7086, and a non-toxigenic genetic mutant, *Microcystis aeruginosa* PCC 7806 Δ*mcyB*. Since its creation in 1997, the Δ*mcyB* strain has been used in comparative physiology studies against the wildtype strain by research labs throughout the world. Some differences in gene expression between what were thought to be otherwise genetically identical strains have appeared due to insertion events in both intra- and intergenic regions. In our Δ*mcyB* isolate, a sulfate transporter gene cluster (*sbp-cysTWA*) showed differential expression from the wildtype, which may have been caused by the insertion of a miniature inverted repeat transposable element (MITE) in the sulfate-binding protein gene (*sbp*). Differences in growth in sulfate-limited media also were also observed between the two isolates. This paper highlights how *Microcystis* strains continue to “evolve” in lab conditions and illustrates the importance of insertion sequences / transposable elements in shaping genomic and physiological differences between *Microcystis* strains thought otherwise identical. This study forces the necessity of knowing the complete genetic background of isolates in comparative physiological experiments, to facilitate the correct conclusions (and caveats) from experiments.

**Supplementary Information:**

The online version contains supplementary material available at 10.1186/s12864-024-10839-5.

## Background

*Microcystis spp.* are potentially toxic bloom-forming cyanobacteria responsible for harmful algal blooms (HABs) around the globe [1]. These cyanobacteria can affect human health and disrupt ecosystems by producing excessive biomass and potentially toxic secondary metabolites, the best known being microcystin [2, 3]. Due to these negative ecological effects, significant research has examined *Microcystis spp.* physiology and ecology to provide insight that may prevent or mitigate HABs [1]. Much of this research has benefited from the global collection of *Microcystis* isolates in labs around the world: model strains that are broadly shared and employed by researchers.

The tools of molecular biology have increased accessibility to sequenced *Microcystis spp.* genomes and made it apparent that *Microcystis* genomes exhibit variability due to abundant transposable elements and repeat regions [4, 5]. In the frequently used lab strain, *Microcystis aeruginosa* PCC 7806, 6.8% of the genome codes for putative transposases [5]. From an ecological perspective, this is an important consideration when investigating *Microcystis* isolates in the laboratory. Mobile elements have been responsible for the loss of the microcystin (*mcy*) gene cluster *via* a major gene deletion event in naturally occurring non-toxigenic strains [6], and the loss of buoyancy in *Microcystis aeruginosa* PCC 7806 has been shown to be mediated by insertion sequences in laboratory conditions [6, 7]. Transposable elements may therefore complicate comparisons between *Microcystis* species/ecotypes, as genes may be present but can change functionality if a transposition event occurs near a gene without disrupting the gene’s architecture. This may be particularly true for experiments in cyanobacterial physiology, where the genomic constancy of cell lines is not necessarily checked thoroughly.

While previous studies have reported on the “plastic” genome of *Microcystis*, studies investigating transposition in *Microcystis* are limited [5, 8]. In culture-based studies, it has been shown that differing nitrogen chemistries and phosphorous concentrations affect the expression of transposases in *Microcystis aeruginosa* NIES 843 [9]. *In situ* metatranscriptome samples collected during a *Microcystis* bloom in western Lake Erie (July - October 2014) revealed gene expression of many strain-specific genes associated with gene plasticity [10]. These studies suggest transposases are transcriptionally persistent and play an important role in the adaptive ecophysiology of *Microcystis* under laboratory conditions and *in situ*.

A recent genome resequencing of the type-strain for *Microcystis aeruginosa* (PCC 7806, the “wildtype” isolate) and a non-toxic mutant thought to have a single insertion of a chloramphenicol resistance cassette in the *mcyB* gene (*Microcystis aeruginosa* PCC 7806 Δ*mcyB*) led to the discovery of chromosome re-arrangements relative to each other [11, 12]. To examine the effects of these changes, we used transcriptome libraries generated from a recent chemostat experiment [13] to examine gene expression under comparable growth conditions. We found multiple genes and gene clusters were differentially expressed between *ΔmcyB* and the PCC 7086 wildtype under identical conditions and concluded some differences in expression were due to endogenous mobile elements. Additionally, we searched for associations between phage-acquired genes and mobile elements within these and other *Microcystis* genomes. Our findings are important caveats for comparative studies of *Microcystis* evolution and ecology, particularly with respect to extrapolation of observations between different labs and from the lab to the field. Our observations confirm that multiple families of mobile elements are active in *Microcystis* and can impact gene regulation when inserted into intergenic and intragenic regions. These observations are particularly salient for efforts that compare wildtype PCC 7806 and Δ*mcyB* for effects of microcystin production, as we demonstrate that there are other differences between these cyanobacterial genomes, which were assumed to be identical, that can influence phenotype.

## Methods

### Strains, DNA extraction & genome sequencing, assembly, and annotation

*M. aeruginosa* strains used in this study were obtained from either the Pasteur Culture Collection (*M. aeruginosa* PCC 7806) or directly from Professor E. Dittmann (University of Potsdam, Germany in 2016), who created the original *Microcystis aeruginosa* PCC 7806 Δ*mcyB* mutant in 1997 [14].We will refer to our *Microcystis aeruginosa* PCC 7806 Δ*mcyB* isolate as Δ*mcyB.* The wildtype isolate, *Microcystis aeruginosa* PCC 7806, which we will refer to as PCC 7806 wildtype, has been in our lab for the last decade and has been maintained as both active cultures and cryopreserved stocks at -150° C. In the current study, we sequenced the genome of *Microcystis aeruginosa* PCC 7806 *ΔmcyB* and re-sequenced the wildtype isolate of *M. aeruginosa* PCC 7806. This provided improved references for transcriptomic analysis (below) which accounted for expression of transposases that were in different positions (or absent) in each genome. For the genome assemblies from *ΔmcyB* (GCA_030553035.1) and wildtype PCC 7806 (accession CP155078), the growth conditions (25 mL batch cultures in modified CT [11], ~ 26° C, ~ 50 µmol photons m^−2^ s^−1^), high-molecular weight DNA extraction, genome sequencing and assembly protocols are as reported in Stark et al. [11]. Genome annotation for the sequenced Δ*mcyB* and our wildtype PCC 7806 was done using NCBI’s prokaryotic genome annotation software (PGAP) [15]. All other genomes used in this paper were downloaded from publicly available datasets at the NCBI’s GenBank [16].

### RNA sequencing

Transcriptional analyses of strains grown in chemostats during warm (26° C) and cold (19° C) temperature treatments was previously reported [13]: these treatments have been shown to alter cellular physiology and biochemistry, including increasing the transcription and biosynthesis of microcystin [13, 17]. The RNA-seq dataset from Stark et al. [13] consisted of 10 time points (two control 26° C, eight 19° C time points). Transcriptomes of duplicate PCC 7806 wildtype and duplicate *ΔmcyB* chemostats were generated, resulting in a total of 40 libraries.

Mapping parameters for all transcriptome libraries were as described in the methods of [13]. Briefly, CLC genomics workbench (v. 23.0.4) was used to map transcriptomic libraries to the *ΔmcyB* (GCA_030553035.1) and the PCC 7806 wildtype (CP155078) genomes. Stringent read mappings were performed using 0.9 length and 0.9 similarity coverage for mapped reads. All reads were normalized by transcripts per million (TPM). We note that the “maximum hits per read” was set to the default of 10, which would make the TPM for some highly repetitive transposases with > 10 copies (IS200 family and ISNYC- family) under-representative of their true expression values. Since increasing the “maximum hits per read” to > 10 resulted in non-specific read-mapping, we decided that keeping the mapping parameters stringent and specific would be preferential. An example of this for IS200 family transposases can be seen in the supplemental materials (Table S1). Differential expression analysis was done as described in [13], using our PCC 7806 wildtype genome. TPM values reported for the sulfate transporter gene cluster in Fig. 5 (*sbp*-*cysTWA*) were from reads mapped to the wildtype PCC 7806 genome (since *sbp* is annotated as two genes in Δ*mcyB)*, all other figures have TPM values that came from mappings to the respective strains (Δ*mcyB* and PCC 7806 wildtype) genome.

### Genome alignments and analyses

Unless noted, default parameters were used for all software. All genomes were aligned using Progressive Mauve (v. 2.4.0) [18]. Alignments of transposase sequences to intergenic regions of interest was done using Clustal Omega [19]. For genes of interest that were annotated as hypothetical or uncharacterized, yet were differentially expressed due to transposon re-arrangement, we ran the translated sequences through PFAM (v. 36.0), HMMER (v. 3.4), and NCBI conserved domain searches to characterize protein domains and predict putative functions [20–25]. BLASTP, BLASTN, and JGI/IMG BLASTN (virus database) were also used to get similarity scores for certain genes/protiens and find homologous proteins/sequences among *Microcystis* strains [23, 26]. To find putative open reading frames for the miniature inverted transposable element (MITE), NCBI ORFfinder was used [23]. ATG-only start codons were considered, and minimal ORF length (nt) was set to 30. The secondary structure of the 187-nt MITE was produced *in silico*, using mFOLD (v. 3.0) [27]. To find putative σ70 promotor regions, we used BPROM [28]. Intergenic regions were used as input, and all search parameters were kept as default.

### MGE Mobile genetic elements (MGE), phage gene(s), and phage-gene discovery

Transposable elements that altered gene expression were discovered by looking at annotated transposases that had a fold change in gene expression ≥ 2 from the differential expression analysis comparing the wildtype and Δ*mcyB* transcriptome libraries [13]. Manual inspection of gene clusters that were differentially expressed at all 10 RNA-seq time points [13] was also done to see if intra- or intergenic mutations were present that were not caused by annotated insertion sequences. To expand our search to include all known insertion sequences in the Δ*mcyB* and wildtype genomes, the ISfinder BLASTn database was used [29]. The e-value was set to 1E-10, and all other search parameters were left as default. Redundancy of any IS alignments was checked by converting coordinate IS outputs to .gff format. This led to the removal of numerous ISCysp15, ISMich1, ISCysp18, ISUnCu10, and ISNpu9 sequences, which were small (400 < bp) and overlapped with ISMae6 and ISMae3 elements in many instances. The ISfinder output did not include IS1634 and *tnpA* (IS200/IS605) which we found through gene expression analysis, so hits > 90% identity to IS1634 and *tnpA* were added into our list of insertion sequences.

To discover genes of putative phage-origin, geNomad [30] was used, along with manual searches (*e.g.*, scanning for genes annotated as “phage”), to screen publicly available complete *Microcystis spp.* genomes from NCBI GenBank (accessed 01/2024) [23, 30]. Default parameters were used for all geNomad runs. Genes with viral hallmark scores of “1” were considered for further analysis using PFAM, nucleotide BLAST, and conserved domain searches to confirm relevant domains/distribution of genes in bacteria.

### PCR validation

To confirm the presence of the MITE in the *sbp* gene that we observed during sequencing, primers were constructed flanking the MITE insertion sequence (forward primer 5’- ACCAAAAAGGTGAGAAGTTAGC-3’, reverse primer 5’- CGAAACCCCACCTTAGCA − 3’) (Figure S1). PCR reaction mix consisted of 12.5 µL EconoTaq Plus Green 2X Master Mix, 1.25 µL of 10 µM forward and reverse primers, 9 µL of molecular grade water, and 1 µl of whole-cell template. The thermocycler program for PCR amplification was set to 95º C for a 5-min initial denaturing cycle. Then, 35 cycles were run with a 95º C / 30 s denaturing, 53º C / 30 s annealing, and 72º C /60 s elongation. A final elongation step was set at 72º C and run for 5 min.

### Reverse transcriptase PCR

Due to the repetitive nature of the MITE in the genome, and the placement of the MITE in the 23S rRNA gene (reads were removed during *in silico* rRNA reduction), we completed a reverse transcriptase PCR to determine if the MITE in the *sbp* gene was transcribed with the gene. Reverse transcriptase PCR was accomplished on RNA samples extracted from chemostat-grown cultures as previously reported [13]. In the present study, we used time points denoted T4 and T10 (Figure S2, S19). All RNA was previously DNAsed with a Turbo DNA-free kit (Invitrogen) and confirmed DNA-free *via* PCR [13]. A Thermoscript Plus RT-PCR kit (Invitrogen) was used to convert isolated RNA to cDNA, using manufacturers protocols. Briefly, each 50 µL reaction had 1 µL of Thermoscript plus/platinum TAQ enzyme, 25 µL of 2X Thermoscript reaction mix, 1 µL of 10 µM forward and reverse primers, 20 µL of DEPC treated water, and 2 µL of ~ 15 ng/µL of template RNA. The thermocycle program for cDNA synthesis was set at 58° C and run for 25 min. Immediately after, a PCR cycle was run, consisting of a 5-minute 95° C incubation period, and 35 cycles of 95° C denaturing for 15 s, 55° C annealing for 30 s, and 72° C elongation for 30 s.

### Sulfate growth assays

To address specific observations from the genomic sequencing and transcriptomics [11, 13], we examined the effect of sulfate availability on cell growth. At the onset of growth assays, we validated that both the *mcyB* chloramphenicol insertion [14] and an observed insertion of a miniature inverted transposable element (MITE) into a *sbp* gene were present in the Δ*mcyB* strain. For sulfate-limited culture conditions, CT medium [11] was reduced to 0.975 µM MgSO_4_, whereas sulfate replete-medium contained 195 µM of MgSO_4_. Cultures were acclimated to ~ 20° C (± 0.4° C) and ~ 50 µmol photons m^−2^ s^−1^ for six days prior to experimentation. Experimental growth temperatures were 19.5° C ( ± 0.3° C) and ~ 50 µmol photons m^−2^ s^−1^. Prior to inoculation for the respective sulfate treatments, all cultures were pelleted and washed twice with sulfate-limited medium. Cells were then inoculated in 25 mL batch cultures of either sulfate-replete or sulfate-limited media. Culture position in incubators underwent daily random re-shuffling to minimize any variance in irradiance. All cultures were grown in triplicate. Cell concentrations were estimated with a Cytoflex Flow Cytometer (Beckman Coulter), with populations gated by red fluorescence (chlorophyll a proxy) and forward scatter (size proxy).

### Protein modeling and alignments of the *sbp* gene products

ColabFold [31] was used create protein models for the wildtype and Δ*mcyB* (MITE insertion) *sbp* gene product. For the input amino acid sequences, the translated products based on the PGAP annotated genes were used. SignalP 6.0 [21] was used to find and remove the signal peptide region for each protein (residues 1 to 24). For each run, the pdb100 database was used as a template, the num_relax was set to 5, and num_recycles was set to 6. All other parameters were kept as default. TM-align [32] was used to align the rank 1 protein models. Default parameters were used, and the alignment was normalized to the smaller (truncated) *sbp* gene product. TM-align scores between 0.5 and 1.0 are in the same fold [32]. Protein alignments were visualized with iCn3D [33, 34].

### Statistical analysis

Normalized RNA-seq libraries (*n* = 40) from a previous chemostat experiment [13] were analyzed with GraphPad Prism (v.8.0.2). For genes of interest, t-tests were done on the normalized TPM values between the *ΔmcyB* and PCC 7806 wildtype strains for two acclimated control time points at 26° C (T1 and T10) and two 19° C acclimated time points (T7 and T8), see Figure S2 for more information. FDR-corrected p-values ≤ 0.05 were considered “significant”.

## Results

### Whole genome alignments of *Microcystis aeruginosa* PCC 7806 and the discovery of a large chromosome inversion

The complete *M. aeruginosa ΔmcyB* genome was 5,103,923 bp long, whereas the wildtype PCC 7806 genome was 5,096,229 bp. These contrasted with the available *Microcystis aeruginosa* PCC 7806SL genome (accession GCA_002095975.1), which is 5,139,339 bp. However, a whole genome alignment of our *ΔmcyB* and PCC 7806 wildtype genome with the published *Microcystis aeruginosa* 7806SL (will be referred to as 7806SL) genome led to the discovery of a duplicated region of 44,534 bp in PCC 7806SL (Figure S3). It is possible the duplicated portion of the 7806SL genome may be due to an assembly error, which only used PacBio RS II long reads for genome assembly [12]. To this end, we decided to not further pursue whole-genome comparisons to 7806SL for this study. Genome annotation and Mauve output data can be found in Table S2.

**Fig. 1:**
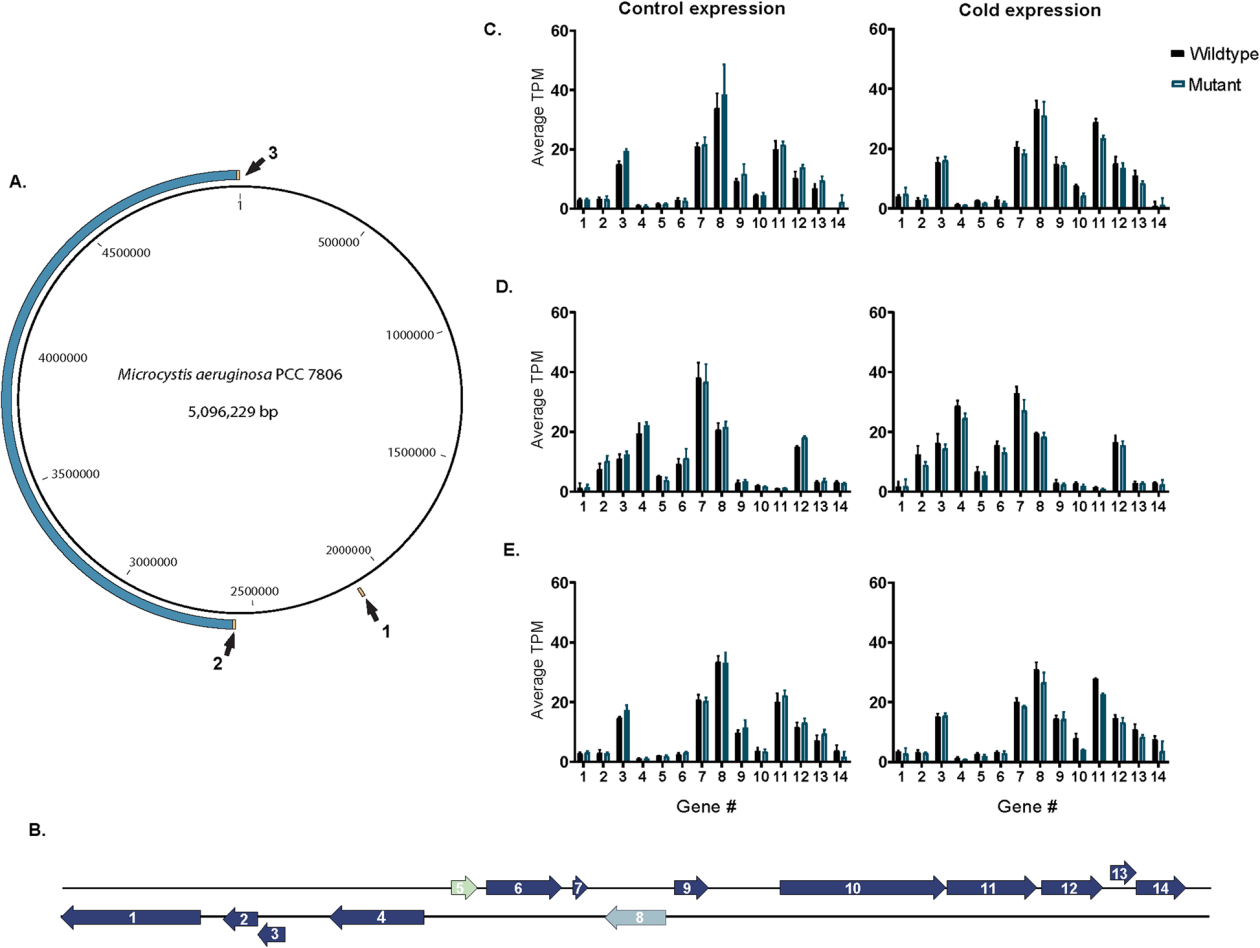
**A** Chromosome map showing the inversion region, in blue of the *Microcystis aeruginosa* PCC 7806 genome from our lab relative to an alignment against the mutant *Microcystis aeruginosa* PCC7806 Δ*mcyB* mutant genome. Orange regions with arrows indicate the locations of a cluster of 14 identical genes, which occur in 3 locations in the genome.​ ​**B** Gene arrangement of the repetitive gene cluster that flanks the chromosome repeat regions in the genomes. Dark blue genes are all uncharacterized proteins, with no homology to known proteins. Genes not colored dark blue have homology to known genes/protein domains. Gene 5 has a Cro/C1 HTH-like protein domain. Gene 8 has phage site-specific integrase/tyrosine recombinase protein domains. ​ ​**C** Gene expression of chromosome repeat region 1 in control (26° C ) (left) and cold (19° C ) (right) growth conditions. ​For average TPM values, *n* = 2 for the wildtype, and *n* = 2 for the mutant. **D** Gene expression of chromosome repeat region 2 in control (26° C) (*n* = 4) and cold (19° C) (right) growth conditions. ​​For average TPM values, *n* = 2 for the wildtype, and *n* = 2 for the mutant. **E** Gene expression of chromosome repeat region 3 in control (26° C) (left) and cold (19° C) (right) growth conditions. ​For average TPM values, *n* = 2 for the wildtype, and *n* = 2 for the mutant

Whole genome alignment of the *ΔmcyB* and wildtype genome uncovered a large chromosome inversion (Fig. 1, Figure S4). The inversion was ~ 2.5 Mbp and flanked on both ends by identical nucleotide sequences ~ 10 kb in length. The ~ 10 kb identical nucleotide sequences encoded a set of 14 genes that existed in 3 copies within both the *ΔmcyB* and PCC 7806 wildtype genome (Fig. 1A, Table S3). Of note, these regions all contained a gene encoding a protein with a cro/C1-type HTH domain, and another gene with tyrosine-type site-specific recombinase/phage integrase domains [20].

We examined the normalized gene expression (TPM) for the 14 genes in the regions flanking the chromosome inversion for control (26° C, *n* = 2) and cool temperature acclimated (19° C, *n* = 2) chemostat transcriptomes (Fig. 1B-C). Most genes (12 out of 14) had TPM values < 30 for both the warm and cold treatments across all three gene clusters (Fig. 1C, D, E). The two most highly expressed genes in these regions (TPM > 30 in some instances) in both *ΔmcyB* and the PCC 7806 wildtype were “gene 7”, which a PFAM search showed has no homology to any known protein domains, and “gene 8”, which is a tyrosine site-specific recombinase/phage-integrase (Fig. 1B). In the wildtype, “gene 7” had average TPM values ranging from 20.49 to 38.13 across the warm and cold treatments. In *ΔmcyB*, average TPM values for “gene 7” ranged from 18.27 to 36.74 across treatments. Gene 8 had average TPM values that ranged from 19.60 to 33.91 for the wildtype and 18.41 to 38.36 for the *ΔmcyB*, across treatments.

### Overview of transposable elements in the Δ*mcyB* and wildtype genomes

Using ISfinder, we found a total of 491 significant hits to numerous families of transposable elements in the *ΔmcyB* genome and 489 significant hits for the wildtype genome. A list of these results can be found in Tables S4, S5 and S6. In total, there were significant hits to 13 different IS-families in both genomes. The IS families that differed in the number of hits were the ISNCY, ISL3, IS1, IS200/605 and IS1634 families (Table S6). We note that our list is likely under representative of the amount of insertion sequences in the genomes, as two transposable elements (IS1634 and IS200/605 families), which altered gene expression, had no hits to the ISfinder database with our search parameters.

### IS200 family transposases

We found multiple copies of a 444-bp IS200/605 family transposase gene in both the wildtype and *ΔmcyB* genomes. In *ΔmcyB*, we found fourteen, where as our wildtype strain had sixteen of these transposases (Table S7, S8). Most of the IS200 family genes have the same genome location between our two strains. In *ΔmcyB*, there was one IS200 family gene whose genomic location differed from the wildtype: it interrupted a solute carrier superfamily (SLC) permease gene (Figure S5). Our wildtype strain had three additional IS200 family transposase genes not found (location wise) in *ΔmcyB*: one interrupted a potassium uptake gene (*trkH*), another interrupted a glycosyltransferase gene, and the third was inserted between a 3-mercaptopyruvate sulfurtransferase and PIN-domain containing protein gene (Figures S6-S8). In *ΔmcyB*, the glycosyltransferase gene was interrupted by an ISNCY-like ISMae2 family transposase instead of a IS200 family transposase (Figure S7).

### IS1-like IMSae3 family transposases

**Fig. 2:**
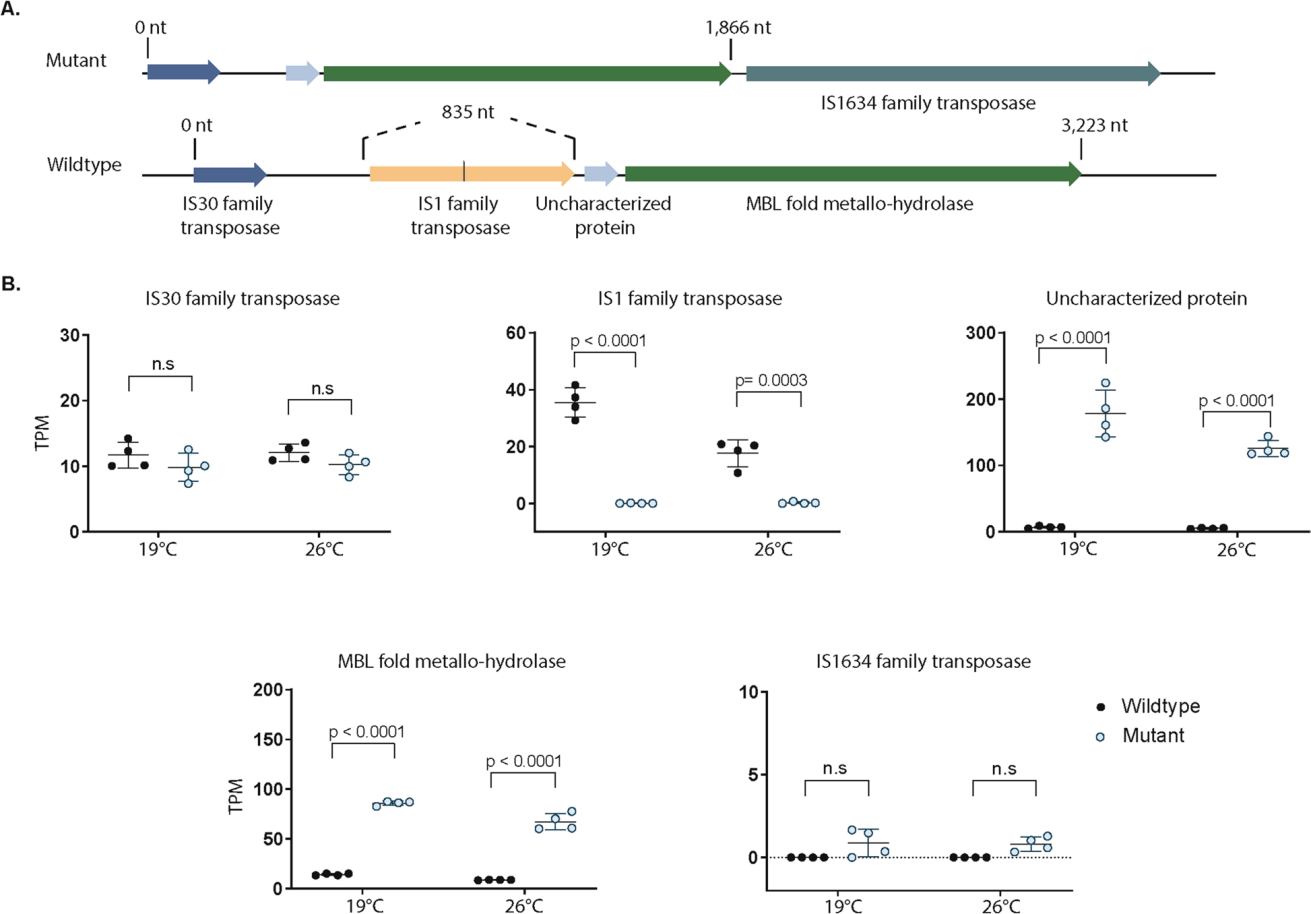
**A** Gene arrangement in Δ*mcyB* versus the PCC 7806 wildtype genome. In the wildtype (bottom arrangement), an IS1-family transposase is inserted before genes encoding an uncharacterized protein and a MBL-fold metallo-hydrolase protein. In Δ*mcyB* (top) the IS1-family transposase is absent from the intergenic region before the uncharacterized protein and MBL-fold metallohydrolase. The insertion of an IS1634 family transposase downstream of the MBL-fold metallo-hydrolase in Δ*mcyB* increases the length of the MBL-fold gene coding region by 80 nt residues. ​**B** Transcription of the genes in the order shown in Fig. 2A. Absence of the IS1-family transposase in the mutant leads to significantly increased expression (*p* < 0.001) of the uncharacterized protein and MBL-fold metallo-hydrolase compared to the expression of these genes in the wildtype, which has the IS1 transposase insertion

Identical IS1-like ISMae3 family transposases (with 737-bp coding regions) were found throughout the *ΔmcyB* and wildtype genomes. The *ΔmcyB* strain had five of such while our wildtype had six (Tables S9, S10). Notably, an IS1-like family transposase situated before an uncharacterized gene and a gene encoding a metallo-hydrolase with a metallo-beta-lactamase (MBL)-fold in the wildtype strain is missing in *ΔmcyB* (Fig. 2A). In the wildtype, the IS1-family transposase is transcriptionally active, showing average TPM values of 17.63 in warm conditions, and 35.53 in cold conditions. Downstream of the transcriptionally active IS1-family transposase in the wildtype we saw significantly reduced expression (*p* < 0.0001, avg. TPM in cold 6.67, in warm 4.7) of a gene encoding an uncharacterized protein, and an MBL-fold metallo-hydrolase (*p* < 0.0001, average TPM 8.55 in warm and 14.06 in cold acclimated) (Fig. 2B). An opposite effect was seen in *ΔmcyB*, where the absence of the IS1-like family transposase results in transcription of the uncharacterized protein (average TPM 125.4 in warm and 178.4 in cold acclimated) and the MBL fold metallo-hydrolase (average TPM 67.12 in warm and 85.78 in cold acclimated). In *ΔmcyB*, there was also an IS1634-family transposase (shows minimal expression) inserted after the MBL-fold hydrolase gene which is not present in the wildtype (Fig. 2B). Insertion of the IS1634-family transposase in *ΔmcyB* increases the length of the MBL-fold gene coding region by 80 nt residues. Based on *in-silico* methods, putative promoter regions were the same for the intergenic regions before the hypothetical protein in the mutant, and before the IS1-transposase in the wildtype (Table S11).

Another gene interrupted by the IS1-transposase was a gene encoding an uncharacterized protein. The gene encoding the uncharacterized protein was situated in-between genes that encode for a MoaD/ThiS family protein and an Acyl-CoA dehydrogenase in *ΔmcyB* (Figure S9). A protein BLAST search of the translated sequence for the uncharacterized protein revealed it had 94% query cover and 32.25% amino acid sequence identity to an Acyl-CoA thioesterase in *Chloroflexota* (TMF61956.1) [23]. Insertion of the IS1 transposase in the gene encoding the uncharacterized protein (putative thioesterase) significantly (*p* < 0.0001) reduced transcription of the uncharacterized protein in *ΔmcyB* compared to the wildtype (Figure S9). The IS1-transposase was transcriptionally active in *ΔmcyB*, with average TPM values of 70.1 in warm and 118.8 in 19°C acclimated conditions. Next to the uncharacterized protein, and present in both the wildtype and *ΔmcyB* genome, was an ISL3-family transposase. The ISL3-family transposase showed minimal expression in Δ*mcyB* compared to the wildtype (all *ΔmcyB* TPM < 5, all wildtype TPM ≥ 13 and ≤ 50) (Figure S9).

**Fig. 3:**
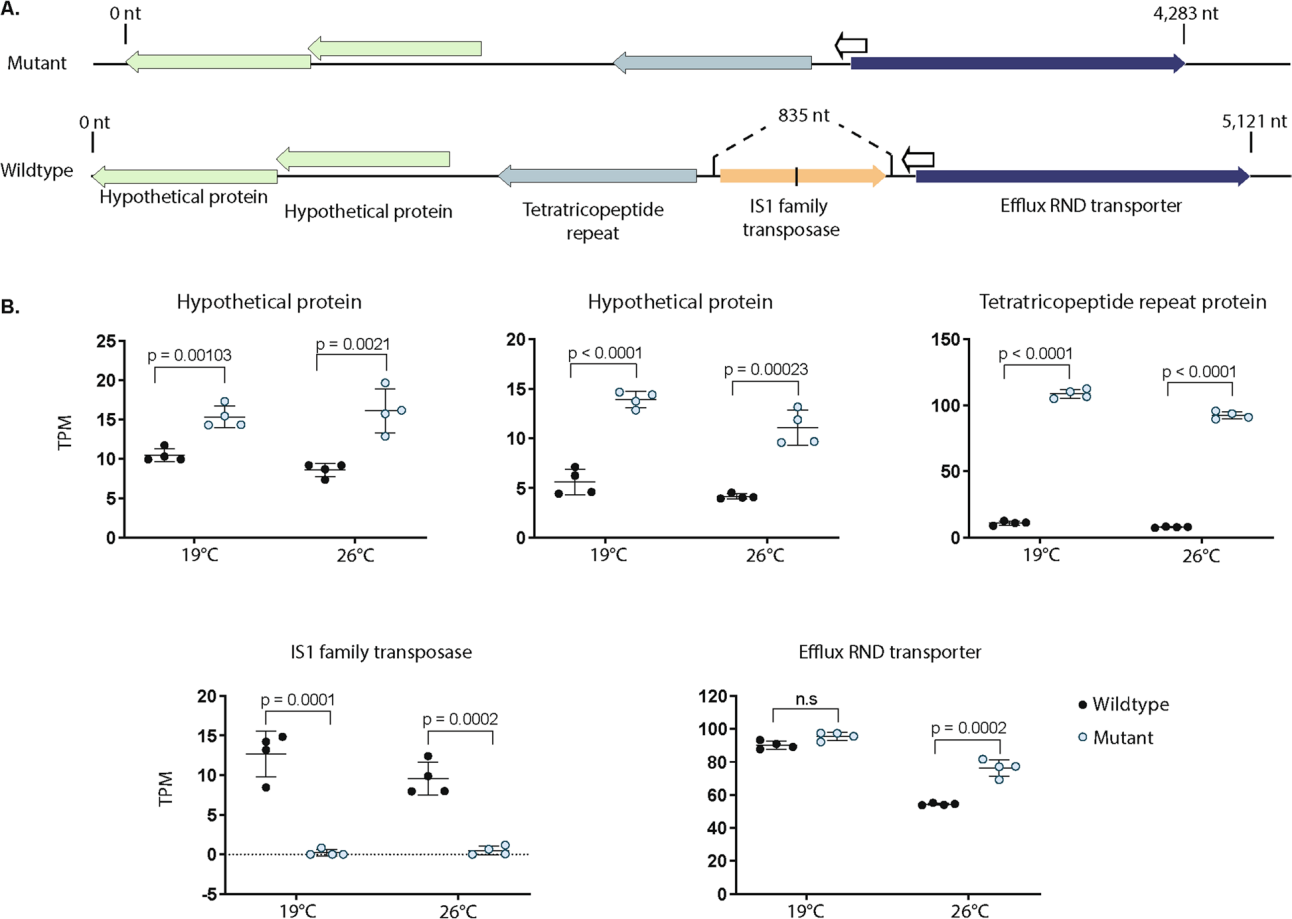
**A** Gene arrangement in Δ*mcyB* (top) versus the PCC 7806 wildtype (bottom) genome. In the PCC7806 wildtype, an IS1-family transposase is inserted in the region between genes encoding a tetratricopeptide repeat protein and an efflux RND transporter. **B** Transcription of the genes in the order shown in Fig. 3A. In the PCC 7806 wildtype, insertion of the IS1-family transposase in the region between genes encoding a tetratricopeptide repeat protein and an efflux RND transporter leads to significantly decreased expression (*p* < 0.001) of the gene encoding a tetratricopeptide repeat protein and a hypothetical protein. Expression of the efflux RND transporter is significantly different between Δ*mcyB* and PCC 7806 wildtype at control growth temperature (26° C) (*p* < 0.001) but not cold (19° C). Differences in expression for the RND transporter are probably not due to the IS1-family transposase, since the two strains have similar expression profiles in the cold. Gene expression data is not shown for the ORF at the end of the RND transporter (white arrow, transcribed on opposite strand) because TPM values were < 6 for both strains and did not appear impacted by the transposase IS1-family

In our wildtype type strain, there was an IS1-like ISMae3 family transposase situated next to a gene that encodes a protein with five tetratricopeptide repeats (TPR) (Fig. 3A) [20, 21]. The presence of the IS1-like ISMae3 transposase led to very low gene expression of the TPR-protein in the wildtype (avg TPM 11.01 at 19° C, 7.90 at 26° C) (Fig. 3B). However, in *ΔmcyB*, the absence of the IS1-like family transposase was accompanied by significantly increased gene expression of the TPR protein (*p* < 0.0001, avg TPM 108.6 at 19° C, 92.46 at 26° C). The two hypothetical proteins located downstream of the TPR-protein also showed significantly higher expression in *ΔmcyB* compared to the wildtype (all *p* ≤ 0.0021), which may mean they were also affected by the position of the IS1- like ISMae3 family transposase. The efflux RND transporter, downstream of the IS1-family transposase had similar expression at 19° C in the *ΔmcyB* and wildtype but showed higher expression in *ΔmcyB* (*p* = 0.0002) at 26° C. No predicted putative promoters were found in the intergenic region before the IS1-transposase in the wildtype. Both strains had the same predicted putative promoters in the intergenic region before the RND efflux transporter (Table S12).

### IS1634 family transposase

**Fig. 4:**
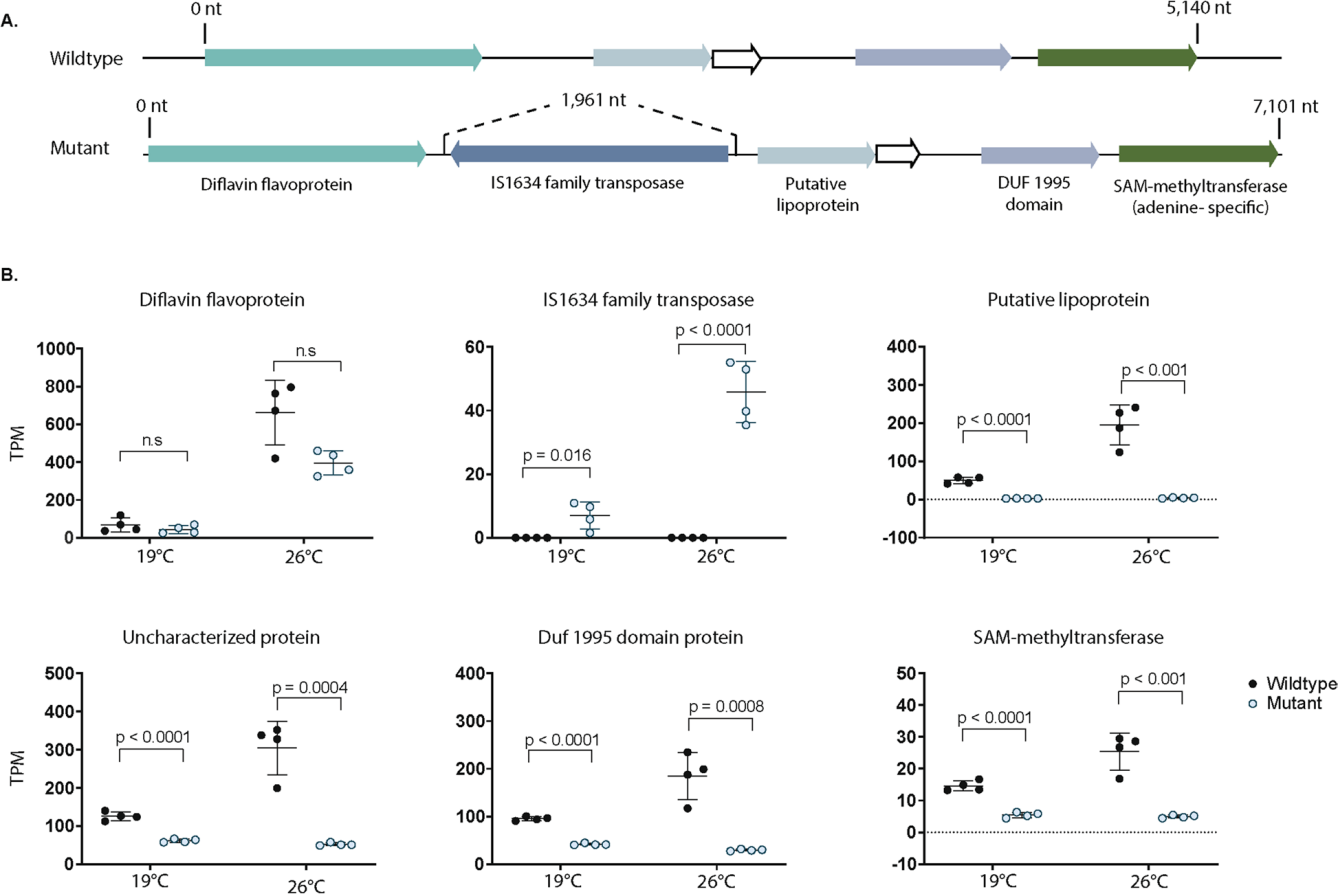
**A** Gene arrangement in PCC 7806 wildtype genome (top) versus the Δ*mcyB* genome (bottom). In Δ*mcyB*, an IS1634 family transposase is inserted between genes encoding a diflavin flavoprotein and a putative lipoprotein. In the PCC 7806 wildtype, this region is absent of the transposase. **B** Transcription of the genes in the order shown in Fig. 4A. In Δ*mcyB*, insertion of the IS1634 family transposase significantly reduced (*p* < 0.001) the expression of four genes, which include a putative lipoprotein, an uncharacterized protein (white arrow), a DUF1995- domain containing protein, and a SAM-methyltransferase.

We found movement/duplication of a 1,746-bp long IS1634 family transposase in the *ΔmcyB* genome. In *ΔmcyB*, ten copies of this specific 1,746-bp long IS1634 family transposase exist, whereas only eight exist in our wildtype strain (Tables S13, S14). In *ΔmcyB*, the IS1634-family transposase is inserted in a putative gene cluster encoding genes with protein homology to a diflavin flavoprotein, a putative lipoprotein, a DUF1995-domain containing protein, and a SAM-dependent adenine specific methyltransferase (Fig. 4A). In *ΔmcyB*, the insertion of the IS1634-family transposase between the diflavin flavoprotein and the lipoprotein resulted in expression of the IS1634-family transposase, which mimics the expression profile of the diflavin flavoprotein (Figure S10). Downstream of the IS1634-family transposase insertion, there was decreased expression (*p* < 0.001) of a putative lipoprotein, a DUF1995 containing protein, and an adenine-specific SAM- methyltransferase gene in *ΔmcyB* (Fig. 4B). There were two putative promoters found in the intergenic regions before and after the IS1634-family transposase, which differed between the mutant and wildtype (Figure S11, Table S15).

### Putative phage-acquired genes affiliated with transposable elements

To investigate whether transposable elements assisted in any gene flow between phage and *Microcystis* genomes, we used geNomad [30] and manual searches for genes annotated as “phage”. The results for all 12 complete *Microcystis* genomes can be found in Table S16. In some cases, we found transposable elements that were putatively phage in origin, and some phage genes situated near transposable elements, or which may be composite transposases. These examples include: a phage tail gene (previously annotated as microcystin related protein B, *mrpB*) that is co-transcribed with a "microcystin related protein A” (*mrpA*) in *Microcystis aeruginosa* PCC 7806 (Fig S12); a “phage protein D”, which has peptidoglycan-binding properties, found in *Microcystis aeruginosa* NIES843 (Figure S13); a phage tail lysozyme and associated peptidoglycan binding/recognition genes in *Microcystis aeruginosa* strains LE3 and NIES298 (Figure S14, Table S17).

### A miniature inverted transposable element (MITE) occurs multiple times in both genomes

**Fig. 5:**
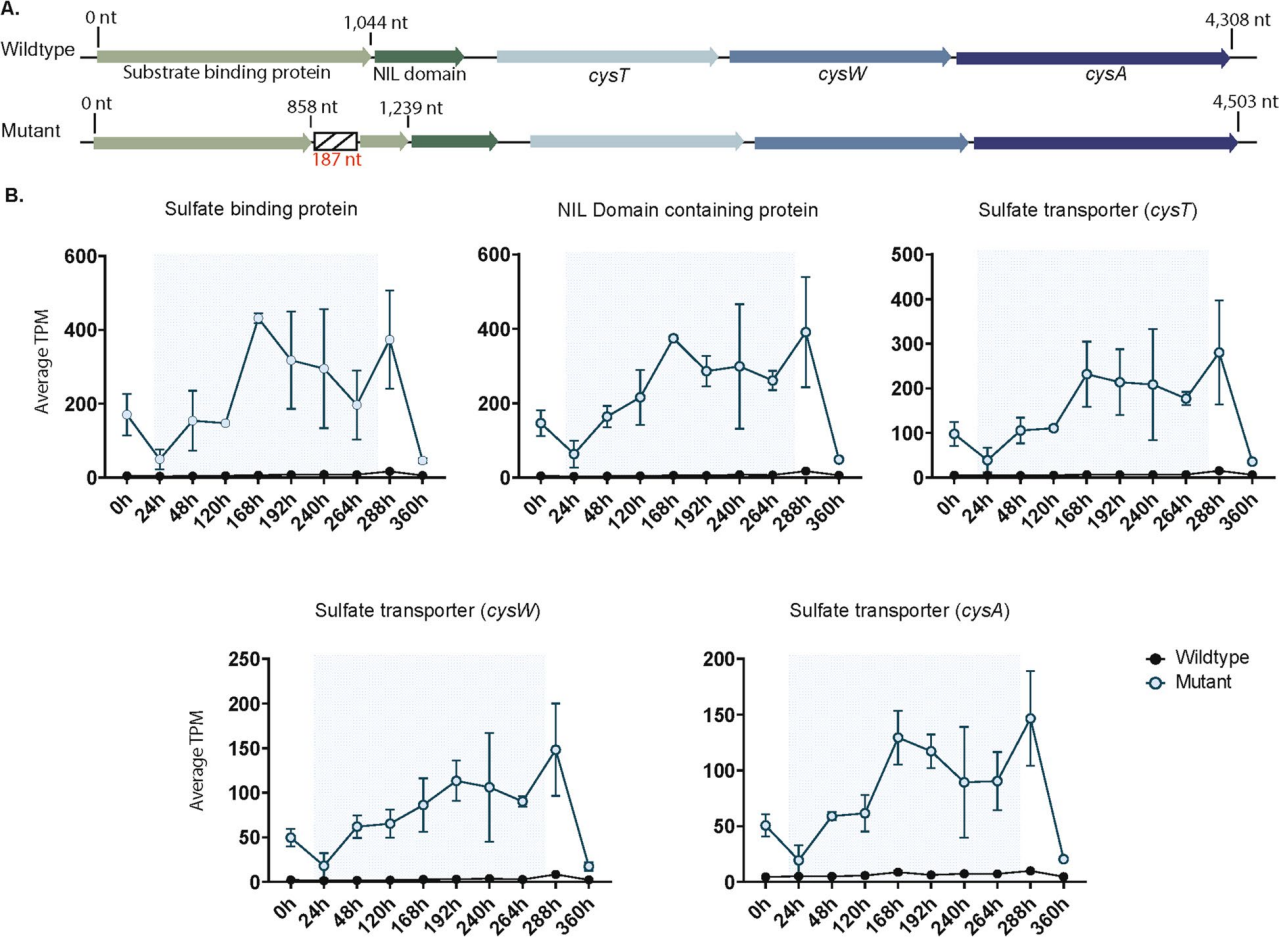
**A** Gene arrangement of the high-affinity sulfate transport gene cluster in *Microcystis aeruginosa* PCC 7806 wildtype (top) and Δ*mcyB* (bottom). Genes are labeled, homologous genes in the mutant are the same colors as those seen in the wildtype arrangement. In the mutant, the sulfate/substrate binding protein gene has a 187-nt long insertion, which is denoted by the striped box interrupting the gene. **B** Average normalized gene expression (normalized by TPM) of the sulfate transporter genes, in order of the gene arrangement seen in Fig. 5A. The open blue circles represent average TPM of Δ*mcyB*, and black circles are the average TPM of the PCC 7806 wildtype. The mutant has a marked differential expression of all five genes, which show fold changes > 2 at most time points. The wildtype shows very little transcriptional activity of any of these genes (all TPM < 20)

 We discovered a 187-bp nucleotide sequence that existed in various regions of the *ΔmcyB* and wildtype genome (Table S18 and S19). The insertion was flanked by direct terminal repeats that were eight nucleotides in length and duplicated from the target sequence, a characteristic of transposable elements [35, 36]. The sequence also has imperfect ITRs nine nucleotides in length (Figure S15). As the insertion sequence did not encode a transposase, we concluded it was a small, non-autonomous transposable element, which are known as miniature inverted repeat transposable elements (MITEs) [37, 38]. While the 187-nt MITEs were located mainly in intergenic regions, often between toxin anti-toxin (TA) systems and transposable elements, in some instances they inserted in protein coding genes (Tables S18, S19). Notably, the MITE is present in one of the two 23S rRNA genes in *ΔmcyB*, our wildtype, and the 7806SL genomes. In the *ΔmcyB* genome, there were thirty-three identical 187-bp long MITEs and thirty-one in our wildtype (Tables S18, S19). The *ΔmcyB* strain had two additional mutations due to this sequence, one located in a gene encoding a sulfate-binding protein (*sbp*) associated with the sulfate transporter gene cluster *cysTWA* (Fig. 5A), and another in a gene encoding the protein LivG (Figure S16).

To assess whether the 187-nt MITE had coding potential, it was run through NCBI ORFfinder: two ORF’s less than 30 amino acids were found [23]. ORF 1 encodes a putative peptide twenty-one amino acids in length (aa sequence MSFSIRVPSLITPVYCIRQTEE), which starts at residue 120 and ends at residue 185 in Frame 3. ORF 2 encoded a peptide eleven amino acids in length (aa sequence MPPYRTITSNEE), starts at residue 38 and ends at residue 3 in Frame 3 (Figure S15).

*In silico* modeling (mFOLD) of the RNA structure of the 187-nt MITE revealed it has a stem-loop structure, which is typical of MITEs (Figure S15) [39, 40]. MITEs are non-autonomous insertion sequences, relying on a transposase to mediate their transposition [37]. To find the family of transposase responsible for MITE transposition, we searched for sequences in the genome that matched the conserved 9-nt long inverted terminal repeat (ITR) of the MITE. Doing so led to the observation that some 1,215-bp long ISL3-family transposases are flanked at both ends by imperfect inverted terminal repeats starting with the sequence 5’-“GGCTCTTCG”-3’, which matches the MITE’s ITRs (Figure S17). Furthermore, the ISL3-family transposases with homologous ITRs are flanked by 8-nt long direct terminal repeats, the same length as the DTRs for the MITE. These characteristics make it likely ISL3-family transposases mediate the transposition of the MITE [37].

A nucleotide BLAST search of the MITE found homologs with > 90% nucleotide identity in twelve other *Microcystis* genomes (Table S20). We also found three *Microcystis* plasmids with homologs of the MITE with ≥ 85% nucleotide identity (Table S21). Additionally, there were three putative viral scaffolds with homologous hits to the MITE, with identities of ~ 86 to 90% (Table S22) [41]. A nucleotide alignment of some of the homologous sequences found in *Microcystis* genomes, all of which had confirmed direct terminal repeats flanking the sequences in the genomes, suggests the 9-nt long imperfect inverted terminal repeat may be a conserved feature of the homologous MITE’s (Figure S15).

### Insertion of the 187-nt long MITE in a sulfate-binding protein gene in the Δ*mcyB* genome

There was differential expression of a sulfate transporter gene cluster *sbp-cysTWA*, which had a > 2-fold increase in gene expression for the *ΔmcyB* isolate (Fig. 5B). The relative expression of *sbp-cysTWA* was highest during cool temperature conditions. In *ΔmcyB*, the gene encoding the sulfate binding protein, *sbp*, had a 187-bp MITE inserted in it. In the wildtype, expression of these sulfate transporters was minimal (TPM < 20). This trend was not observed for the disrupted *livG* gene in *ΔmcyB*, where average TPM values for the gene regions flanking the MITE were like those of the wildtype (Figure S16).

**Fig. 6:**
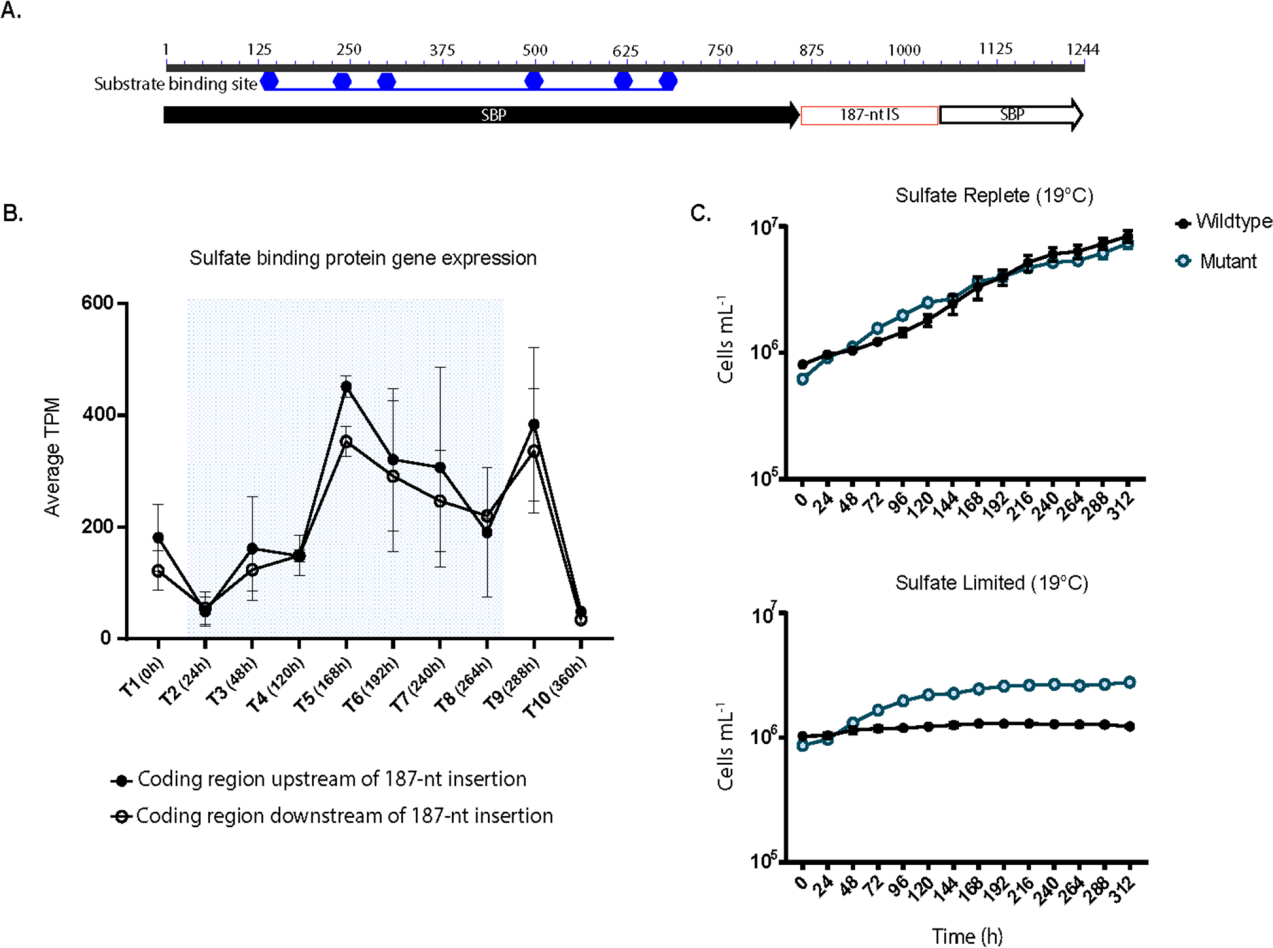
**A** *In-silico* determination of the substrate binding site conserved residues of the sulfate binding protein gene in the Δ*mcyB* strain, based on and adapted from the output of NCBI’s conserved domain search [23, 25]. The 187-nt insertion sequence (IS) is located downstream from the substrate binding domain. **B** Gene expression profile of the sulfate binding protein coding regions upstream and downstream of the MITE insertion sequence in the Δ*mcyB* strain. The 187-nt long MITE does not appear to affect the expression of the gene downstream of the insertion sequence in the Δ*mcyB* strain. **C** Growth assays of the PCC 7806 wildtype and Δ*mcyB* strain in sulfate replete, or sulfate limited media, grown at 19° C. The mutant Δ*mcyB* strain appears to have a growth advantage in sulfate limited media when grown at cold temperature compared to the PCC 7806 wildtype. The PCC 7806 wildtype barely grew above the initial inoculum of (avg. starting ~ 1.03 x10^6^, avg. ending ~ 1.23 x 10^6^), whereas the Δ*mcyB* starting inoculum was (avg. ~8.65 x 10^5^), and it reached maximum cell concentrations of (avg. ~2.8 x 10^6^)

For the *sbp* nucleotide sequence, the conserved residues of the substrate binding site occur from residues 136 to 678, upstream of the MITE insertion sequence, which occurs from residues 859–1053 [23] (Fig. 6A). The translated protein sequence for the wildtype *sbp* gene is 347 aa in length. The Δ*mcyB sbp* gene is split in two due to the MITE, and codes for proteins 286 aa and 64 aa in length. Despite the mutated *sbp* protein product being truncated, the amino acid sequences for the MITE-inserted *sbp* gene still share identical amino acid residues with the wildtype sbp protein (Figure S18). This means that the 286 aa-long mutated *sbp* shares the same conserved amino acid residues needed for sulfate binding. Protein structure alignments of the 347-aa and 286-aa long *sbp* genes also showed similar folding of the two proteins with a TM-align score of ~ 0.986 and an RMSD of 0.79 when normalized to the smaller protein (Figure S18).

To understand whether the MITE insertion interrupted transcription of the *sbp* gene, we analyzed the normalized (TPM) reads for the altered *sbp* gene (upstream and downstream of the insertion). Reads consistently mapped to the region of the gene before the MITE insertion and the region of the gene after the MITE among all *ΔmcyB* transcriptome libraries (Fig. 6B). Due to the repetitive nature of the MITE in the genome, we could not determine normalized gene expression of the MITE at specific sites, including in the *sbp* gene. However, reverse transcriptase PCR confirmed the MITE is transcribed with the gene (Figure S19). For the wildtype, we observed similar sized transcripts (189-bp in length) in the RT-PCR (cDNA) reaction and the gDNA-PCR reaction. For *ΔmcyB*, RT-PCR amplified faint bands 376-bp in length, signifying that the *sbp* gene was transcribed through the MITE in *ΔmcyB* (Figure S19).

We assessed whether the increased gene expression of the *sbp*-*cysTWA* gene cluster in the *ΔmcyB* isolate allowed for improved growth in a sulfate-limited environment compared to the wildtype (Fig. 6C). *ΔmcyB* and the wildtype showed similar growth when grown in sulfate-replete conditions at ~19.5˚ C (Fig. 6C). In sulfate-limited conditions, the *ΔmcyB* strain reached higher cell density than the wildtype (Fig. 6C).

A homologous gene search for other *sbp* genes in *ΔmcyB* and the wildtype turned up with a match to a 1,083-nt long *sbp* gene. This gene was not associated with a *cysTWA* permease complex. Protein alignments of the two *sbp* gene products showed they shared 96% coverage with 59% (205/345) of the amino acid residues having identical matches. The aa sequence from the homologous *sbp* gene did not have a signal peptide region like the *sbp* (*cysTWA* associated) gene. This other *sbp* gene was also differentially expressed between *ΔmcyB* and the wildtype, where average Δ*mcyB *expression had average TPM values of ~ 350 at some time points, whereas the TPM values for the wildtype did not exceed 30 (Table S23).

## Discussion

In this study we observed mobile genetic features of *Microcystis aeruginosa* PCC 7806 *via* comparison to a Δ*mcyB* mutant strain generated ~ 26 years ago. Some changes in gene expression were caused by mobile elements, which include IS200/IS605, IS1-like, ISNCY and IS1634 family transposases. Genetic changes were also caused by a 187-nt long MITE, which is likely transposed by an ISL3-type transposase. Three of these families of transposases (IS1-like, IS1634, and ISL3) are DDE-type dsDNA transposons, whose catalytic triad (Asp, Asp, and Glu) use Mg^+^ or Mn^+^ as cofactors to facilitate transposition [42, 43]. IS200 transposases are part of the HuH-enzyme superfamily, and work on ssDNA through a “peel and paste” mechanism [44]. In some instances, as is the case for IS200 transposases, transposons can produce regulatory sRNAs, having widespread effects on gene expression [45]. Some transposon-produced sRNAs can also function as dual regulators, effecting host and transposon gene expression [46]. The RNA-binding protein, Hfq, is also able to post-transcriptionally inhibit different families of transposases [47, 48]. Aside from the various regulatory mechanisms inhibiting transposition, it is also thought that transposition activity is maintained at a low-level in cells due to deleterious mutations [49–51]. However, in this paper, we confirmed through gene expression data and genomic comparisons, that these five families of transposable elements are active in *Microcystis* and altered gene expression by insertion in both inter and intra-genic regions. Movement of transposable elements and the resulting phenotypic changes from these events may play a role in producing the contradictory results found in the literature with respect to the influence of different environmental variables, *Microcystis* physiology, and microcystin production [52]. While natural strain variability already complicates *Microcystis* work, the effects of transposable elements on *Microcystis* genomes creates further confusion as to how to characterize *Microcystis* “strains” to make *Microcystis* research consistent and reproducible from lab-to-lab.

### Chromosome inversion

A major difference between the *ΔmcyB* and wildtype genome was a chromosome inversion, roughly 2.5 Mbp in length. In *Staphylococcus aureus*, chromosome inversions are reversible, and have been shown to result in phenotype switching and prophage activation [53, 54]. Of the 14 genes flanking both ends of the inversion region, only two had identifiable protein domains. These included a gene that encodes a protein with a Cro/C1-type HTH domain and a tyrosine-type site-specific recombinase/phage integrase (which is one of the most highly expressed genes in the cluster). Cro/C1-type HTH domains are involved in site-specific DNA binding and are well characterized as lytic/lysogenic switches in bacteriophage but have been shown to have roles in bacteria as well [55, 56]. Tyrosine site-specific recombinases also have numerous roles, where they facilitate the rearrangement (which includes integration/excision and inversion) of DNA molecules [57]. While large chromosome inversions have been reported and shown to impact genes in cyanobacteria before, they have not yet been described in *Microcystis* [58, 59]. Unfortunately, a limitation of this study is the uncertainty of when this inversion arose. We currently cannot say whether it was naturally occurring, or the result of the genetics work done on the Δ*mcyB* genome. Like so many other strains of microorganisms, this strain has been passaged, transferred and shared many times throughout the global community interested in studying the bloom-forming organisms. To this end it is possible there may exist strains of the mutant that do not have this inversion: this and the other observations we make serve as a caution to researchers studying this globally pervasive bloom former.

### Gene activation and silencing by transposases

We found five families of transposases whose insertion into intragenic or intergenic regions resulted in the differential expression of nearby genes in Δ*mcyB* and the wildtype. These include the IS1-like ISMae3 family transposases, and IS1634 family transposases. In one instance in *ΔmcyB*, the absence of an IS1 family transposase results in the transcription of an MBL-fold metallo-hydrolase gene (Fig. 2). In another instance, an IS1 transposase was located by a gene encoding a putative TPR-protein in the wildtype, which resulted in differential gene expression of the TPR-protein and three hypothetical/uncharacterized genes (Fig. 3). The gene encoding the MBL-fold metallo-hydrolase has homology to RNA-processing exonucleases, which may indicate it has RNAse activity [60]. Tetratricopeptide repeat proteins mediate protein-protein interactions and can be involved in various functions including transcription and formation of multi-protein complexes [61, 62]. While it has previously been shown that this IS1-like ISMae3 family transposase inserted in the gas vesicle gene, *gvpV*, transcriptionally inactivating it in *Microcystis aeruginosa* PCC 7806 [7, 63], here we show that IS1-like elements can also inactivate nearby genes by inserting in intergenic regions.

IS1634 transposases also appeared to be mobile, and lead to gene silencing/decreased expression of a putative gene cluster of unknown function in *ΔmcyB*. Insertion of a IS1634 transposase in the intergenic region between a diflavin flavoprotein and a putative lipoprotein led to silencing of the putative lipoprotein, and decreased expression of genes downstream of the lipoprotein, including a SAM-methyltransferase. Intragenic transposition events like we see with IS1634 and IS1-family transposases could complicate genomic comparisons, as these genes are present in both strains, yet are silenced/have significantly reduced expression due to intergenic insertion.

### MITES are abundant across *Microcystis* genomes

While *Microcystis* genomes have been suggested to be “plastic” due to the abundant transposases, there has not been much focus on the role of MITE’s in *Microcystis*, aside from a couple of studies [4, 5, 8]. MITE’s are short (usually < 500 bp in length) non-coding sequences with high copy numbers that are mobilized in the genome *via* transposases [64]. We found that the 187-long MITE in the *sbp* gene had imperfect inverted terminal repeats that matched those of ISL3-family transposases. It also had a stem-loop secondary RNA structure, another characteristic of MITES [39]. One exception we found was that that *in silico* analysis of the 187-bp MITE suggested it has two putative open reading frames overlaying the inverted terminal repeats, which is uncommon for MITEs [65]. MITE homologs were also present in other *Microcystis* strains, plasmids, and putative *Microcystis* virus sequences. MITE’s have previously been described in *Microcystis aeruginosa* NIES-843 and FACHB-843 and have been shown to be present in *Microcystis* CRISPR sequences [4, 66, 67]. While it’s apparent MITEs are widespread in *Microcystis*, MITEs’ effect on gene expression in *Microcystis* has not been explored. This is important, as MITEs have been shown to influence gene expression in eukaryotes and prokaryotes through production of miRNA’s, regulatory motifs, and promotor propagation [38, 65, 68]. Additionally, transposition of the MITE into one of the 23S rRNA genes could have widespread phenotypic effects. The 23S rRNA gene product functions as a ribozyme that catalyzes the peptidyl transferase step of protein synthesis [69]. Mutations in the 23S rRNA gene have been shown to confer antibiotic resistance in bacteria [69]. What roles the MITE could play in the functionality of the mutated 23S rRNA gene or its product needs further lab validation to see if it offers phenotypic advantages/disadvantages.

### Can MITEs alter gene expression in *Microcystis aeruginosa* PCC 7806?

One striking difference between *ΔmcyB* and the wildtype strain transcriptomes was the differential expression of the sulfate transporter gene cluster, *sbp*-*cysTWA.* All genes in this cluster were identical between the two strains (at a sequence level) except for the presence of a MITE inserted in the *sbp* gene in the *ΔmcyB* isolate downstream of putative conserved ligand-binding residues. While the conserved sulfate-binding residues were still present in the translated protein product for the mutated *sbp* gene, we are unsure if this would result in a functional protein. Studies in other cyanobacteria have shown the activity of *sbpA*-*cysTWA* is regulated by sulfate availability [70, 71]. In *Synechococcus sp.* strain PCC 7942, it has been shown that *sbpA*-deficient mutants are able to grow on sulfate, and only show differences in phenotype from wildtype cells when grown on sulfate-limited medium [70]. Instead, it appears that the *cysT*, *cysW*, and *cysA* genes are essential for sulfate uptake [70]. Therefore, the functionality of *sbp* in this gene cluster may not be a concern for *ΔmcyB*, depending on the growth conditions. Based on our growth assays in sulfate-replete and sulfate-limited media, *ΔmcyB* did appear to have a slight growth advantage over the wildtype in sulfate-limited conditions, as the wildtype was unable to grow beyond the initial inoculum. However, we are unsure if the wildtype’s inability to grow to the same density as Δ*mcyB* in sulfate-limited cold growth conditions is due to differences in the *sbp*-*cysTWA* operon, or if it is related to microcystin production, which is reliant on sulfur [72].

As *sbp-cysTWA* has been shown to be regulated by sulfate availability, this implies the MITE insertion in the *sbp* gene is influencing the activity of *sbp*-*cysTWA* in *ΔmcyB* in some capacity, or *ΔmcyB* has a higher demand for sulfur then the wildtype. It could also be the case that both scenarios are true. We did find another *sbp* gene in the genome. This other *sbp* gene shared 59% amino acid identity with *sbp* (*cysTWA* associated), did not have a signal peptide, and was not associated with other sulfate transporter *cysTWA* genes. However, the homologous *sbp* gene was differentially expressed in *ΔmcyB* versus the wildtype. Whether this other *sbp* gene is upregulated in *ΔmcyB* to compensate for the disrupted *sbp-cysTWA* associated gene (if it is unfunctional), or if this is a differential stress response in *ΔmcyB*, we cannot definitively determine. Unfortunately, to confirm the MITE’s role in the differential expression of *sbp-cysTWA*, it would need to be removed from *sbp* in *ΔmcyB*, a task not presently tractable in a non-model organism like *Microcystis*, which has abundant endonucleases that interfere with genetics [14, 73]. Regardless, our genomic comparisons suggest there is active transposition of MITE’s in *Microcystis*, and future work should be done to understand how they may contribute to gene expression/regulation, especially because they can be easily overlooked if annotated genome features are only considered. Additionally, if regulation of this gene cluster is the same in *Microcystis* as other model cyanobacteria, and expression is not influenced by the presence of the MITE, it would imply *ΔmcyB* requires more sulfur then the wildtype [70, 72].

### Are there other roles for transposable elements in *Microcystis* genomes?

While we have illustrated how transposases have contributed to genetic and physiological changes in the commonly used *ΔmcyB* and wildtype PCC 7806 strains in batch culture, we also wondered if transposable elements may serve other roles. A specific question that arose was the role of these elements in the horizontal transfer of genetic material from phage to the host cell. While we have not addressed this conclusively, we did observe some putative phage-related genes in PCC 7806 situated near transposases. In the *Microcystis aeruginosa* PCC 7806 genome, there is a gene encoding a “microcystin related protein A” (*mrpA*) associated with a phage tail gene, which was previously annotated as *mrpB* (microcystin related protein B). These two “microcystin related proteins”, of putative phage origin, are transcribed together in both the wildtype and Δ*mcyB*, but have higher expression in *ΔmcyB* at one time point in our RNA-seq dataset (Fig. S11). The two genes, *mrpA* and *mrpB* (phage tail), are situated in-between IS4 and ISNCY-family transposases and a ribonuclease type II BrnT/BrnA toxin anti-toxin (TA) system [74]. While we could not determine the normalized gene expression of this ISNCY-family transposase due to repetitive copies of this gene in the genome, we found through a nucleotide BLAST search that it has previously been reported as “ORF3” in a study dating back to 2001 [75]. This indicates these three genes have likely been present in this gene array for > 20 years.

Although we found a few examples of putative phage genes situated near transposable elements, it cannot be definitively determined that transposition events facilitated gene acquisition. As shown in several examples in this paper, transposable elements may be activating or inactivating these putative phage-acquired genes and could be there by chance. However, the acquisition of these putative phage genes and the roles they may play in the cell would be an interesting route to pursue, as they could have implications for HAB research. Some putative phage acquired genes seen in the *Microcystis spp.* genomes have the ability to self-propagate (group I intron endonucleases and group II intron reverse transcriptases). Thus, it seems possible that phage-acquired genes may themselves be autonomous elements, with the ability to contribute to genetic/physiological changes in *Microcystis* genomes.

## Conclusions

We compared the genomes of *Microcystis aeruginosa* PCC 7806 and it’s mutant PCC 7806 *ΔmcyB*, the latter of which was created over 25 years ago. Since the creation of *ΔmcyB*, there has been no genetic comparison between *ΔmcyB* and the wildtype. Our work has revealed the activation/silencing/differential expression of certain gene clusters in the two strains, mediated by IS1, ISL3, IS1634, ISNCY and IS200 type transposable elements. Transposition events are thought to be rare as transposition has been shown to be tightly regulated [46, 49], however as we and others have shown, this may not be the case in *Microcystis* [7]. As transposases can be activated by specific stressors, future work should focus on the activators of various families of transposable elements in *Microcystis*, and how each might result in genomic re-arrangements [76]. Insertion of transposases both inter- and intragenically can complicate genomic and physiological comparisons of *Microcystis* strains and interfere with the reproducibility of experiments. Active transposition in *Microcystis* may in part explain contradictory studies which investigate abiotic effects on *Microcystis* physiology and microcystin production. Because of this, we suggest labs need to routinely re-sequence strains to account for phenotypic variability, and work to maintain the genomic stability of their stocks through cryopreservation. As these mutations have been compounded over the last 25 years of strain maintenance in culture, the activation/silencing of some of these gene clusters may also provide additional insight into microcystin’s intracellular role.

## Supplementary Information

Supplementary Material 1.

Supplementary Material 2.

## Data Availability

Cultures are available as described in the methods section. The *Microcystis aeruginosa *PCC 7806 wildtype genome is available on NCBI GenBank under Bioproject PRJNA1104278, accession CP155078. The genome of the *ΔmcyB* isolate was previously published on NCBI GenBank under GCA_030553035.1. All transcriptomes were published previously and are available in the SRA database under the Bioproject PRJNA1008692.

## Abbreviations

MITE: miniature inverted repeat transposable element
Mbp: megabase pair
ITR: Inverted terminal repeat
ORF: open reading frame
HAB: harmful algal bloom
