# Supplementary material for: Mobilome impacts on physiology in the widely used non-toxic mutant Microcystis aeruginosa PCC 7806 ΔmcyB and toxic wildtype
Source: BMC Genomics. 2024 Oct 3;25:922. doi: 10.1186/s12864-024-10839-5 (PMC11448079; doi:10.1186/s12864-024-10839-5)
Supplement: Supplementary file 1 — Supplementary Material 1. [file 12864_2024_10839_MOESM1_ESM.pdf]

Supplemental Figures for
“Mobilome impacts on physiology in the widely used non-toxic mutant *Microcystis aeruginosa* PCC 7806 Δ*mcyB* and toxic wildtype”

Gwendolyn F. Stark[1], Alexander R. Truchon[1], Steven W. Wilhelm[1*]

[1] University of Tennessee Knoxville, Department of Microbiology, USA

*author for correspondence: wilhelm@utk.edu

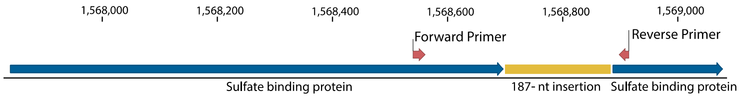

Figure S1: Layout of the gene encoding a sulfate binding protein in the *Microcystis aeruginosa* PCC 7806 Δ*mcyB* strain. Blue regions are coding sequences for the sulfate binding gene (*sbp*). In orange is the 187-nt MITE, located from 1568698 to 1568884 in the Δ*mcyB* genome. Primers were made to amplify a 181 bp region in the PCC 7806 wildtype (no insertion present), and a 376 bp region in Δ*mcyB*.

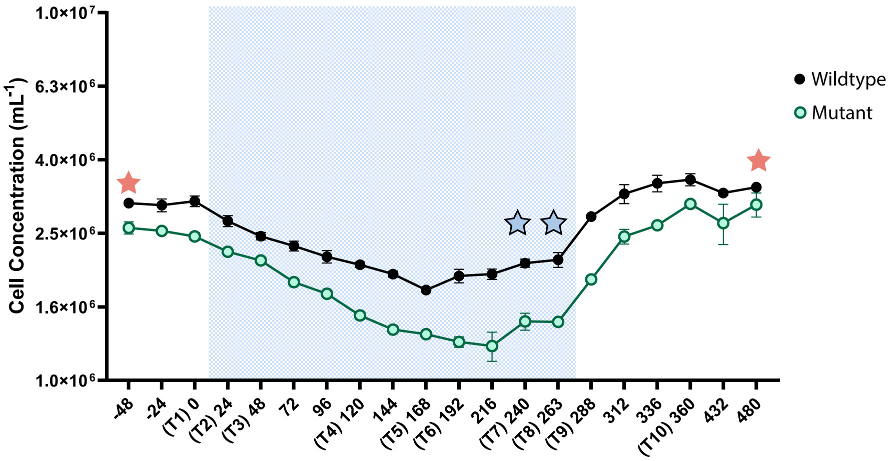

Figure S2: Cell concentrations (y-axis) of a chemostat experiment done for a previous manuscript by Stark et al. [1].Time in hours is on the x-axis. Normalized gene expression between the *Microcystis aeruginosa* PCC7806 Δ*mcyB* and *Microcystis aeruginosa* PCC7806 wildtype strains were compared for the cold (19° C) treatment (T7 and T8, blue stars), and control "warm" (26° C) treatment (T1 and T10, orange stars).

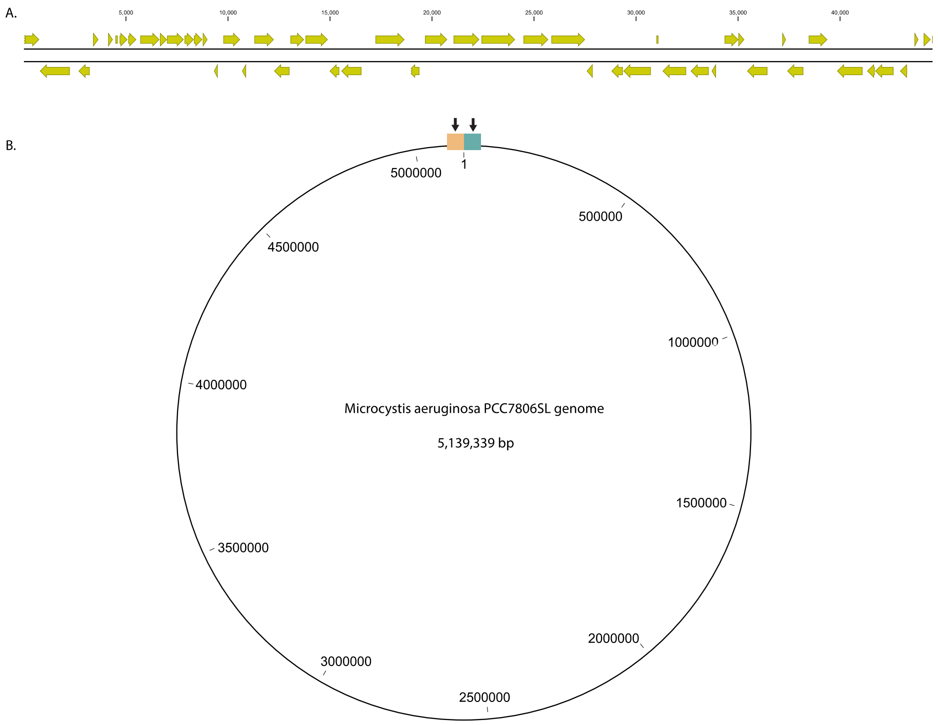

Figure S3: A) Duplicated region of the *Microcystis aeruginosa* PCC 7806SL genome downloaded off NCBI's GenBank. The region is ~45 kb in size, and codes ~49 genes. The region located from 5094806 to 5139339 codes a transposase which is not seen in the duplicated region from 1 to 43125. B) Genome map of *Microcystis aeruginosa* PCC 7806SL. Duplicated regions of the genome (seen above) are denoted by the orange and the green box. The orange box represents the region that has an additional transposase in the region.

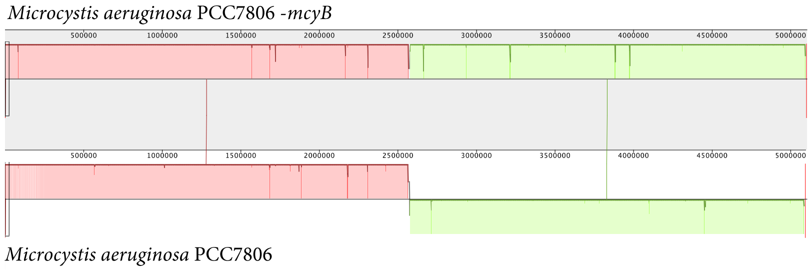

Figure S4: Mauve whole-genome alignment of our *Microcystis aeruginosa* PCC 7806 wildtype (bottom) and Δ*mcyB* (top) strains. Genome regions that are the same color correspond to one another. In green is the region of the genome that is inverted between the PCC 7806 wildtype and Δ*mcyB* genome.

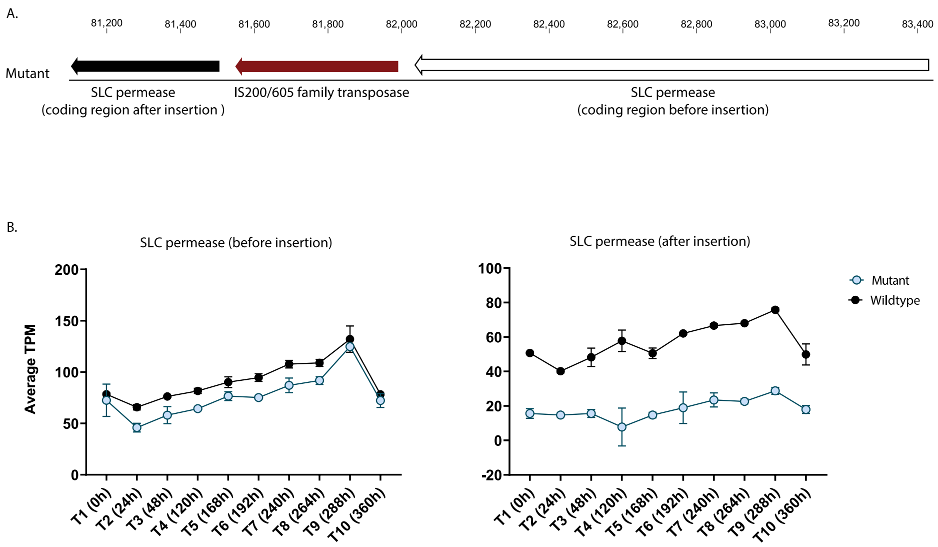

Figure S5: A) A SLC permease gene was interrupted in the *Microcystis aeruginosa* PCC 7806 Δ*mcyB* strain by a IS200/605 family transposase. B) The gene expression of the coding region of the SLC permease before the transposase is similar to the gene expression profile seen in the PCC 7806 wildtype, however, transcripts for the coding region of the gene after the insertion are about half that of the wildtype.

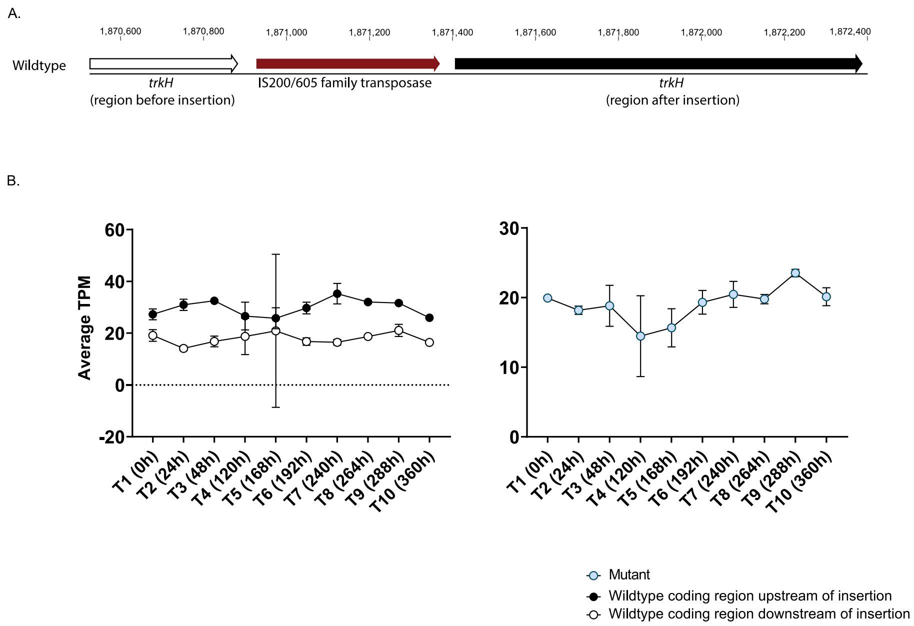

Figure S6: A) A IS1200/605 family transposase is present in the *Microcystis aeruginosa* PCC 7806 wildtype genome, interrupting a *trkH* gene. The *trkH* gene is not interrupted in the Δ*mcyB* genome. The mutation does not appear to cause a significant difference in expression from the *Microcystis aeruginosa* PCC 7806 Δ*mcyB* genome.

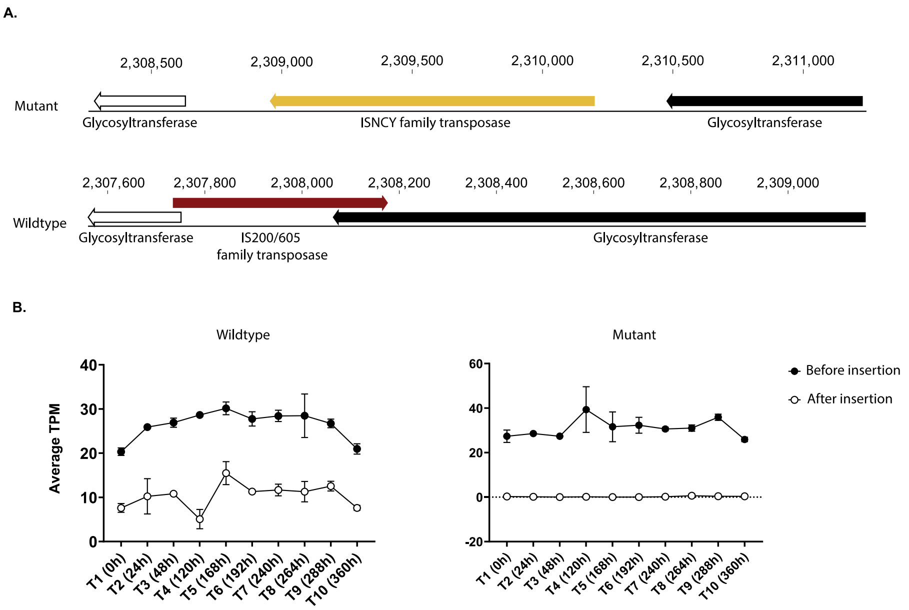

Figure S7: A) A glycosyltransferase gene in the *Microcystis aeruginosa* PCC 7806 Δ*mcyB* and PCC 7806 wildtype genome was interrupted in both strains, by two different types of transposases. In Δ*mcyB*, the glycosyltransferase gene was interrupted by a ISNCY (insertion sequence not characterized yet) transposase, whereas in the wildtype the glycosyltransferase gene was interrupted by a IS200/605 family transposase. B) Gene expression of the interrupted glycosyltransferase gene in Δ*mcyB* and the PCC 7806 wildtype. In the wildtype, the IS200/605 insertion reduced transcript abundance by approximately half for the region of the gene after the insertion sequence. In Δ*mcyB*, insertion of the ISNCY transposase in the middle of the gene resulted in no expression for the coding region after the insertion.

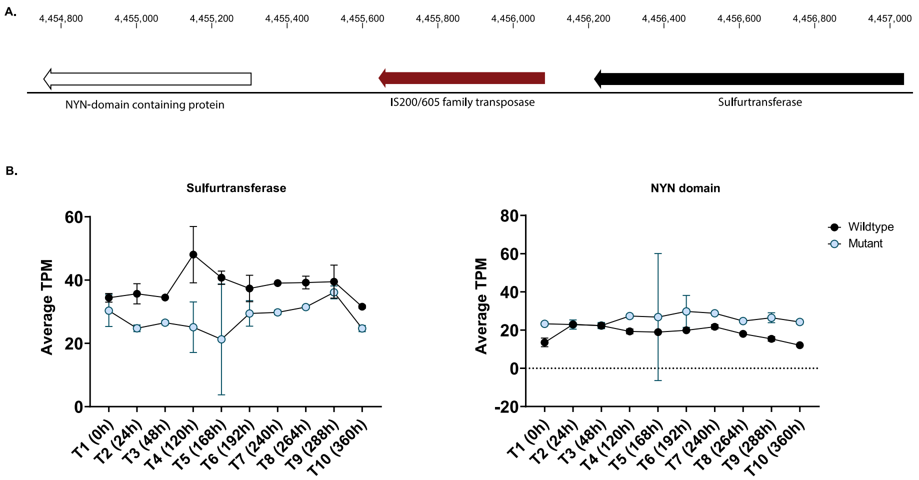

Figure S8: A) A IS200/605 family transposase inserted between genes coding for a sulfurtransferase and a NYN-domain protein in the *Microcystis aeruginosa* PCC 7806 wildtype strain. B) Insertion of the transposase did not seem to effect gene expression of the gene encoding a NYN-domain containing protein in the PCC 7806 wildtype, as the expression profile was similar to that of the PCC7806 Δ*mcyB* strain.

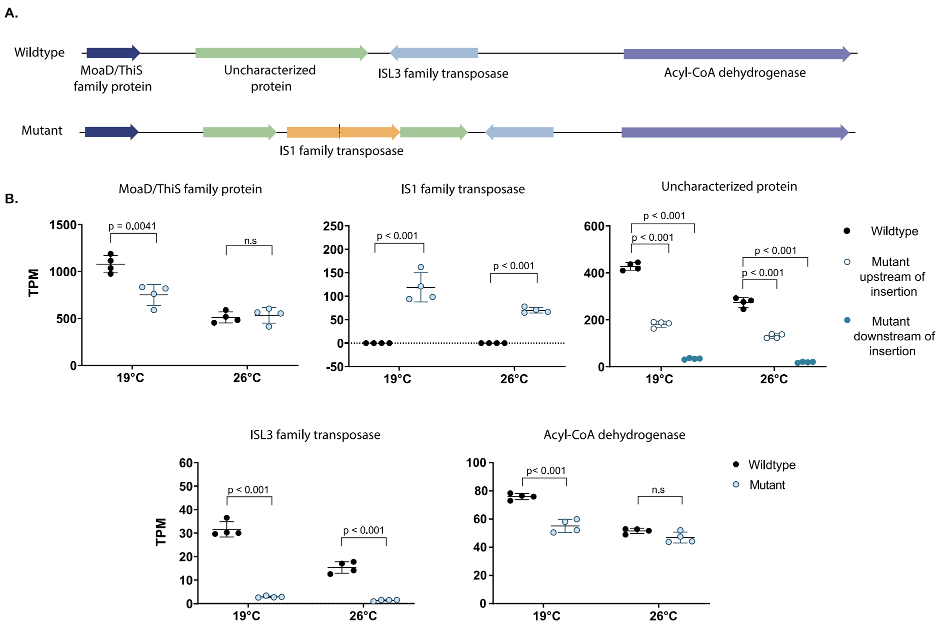

Figure S9 : A) Gene arrangement in *Microcystis aeruginosa* PCC 7806 wildtype (top) versus the *Microcystis aeruginosa* PCC 7806 Δ*mcyB* (bottom) genome. The gene arrangement shows where a IS1-family transposase is inserted in a gene encoding an uncharacterized protein (putative thioesterase) in Δ*mcyB*, and genes surrounding the mutated gene. The PCC 7806 wildtype arrangement shows the same hypothetical protein and gene area, unmutated. B) Transcription of the genes in the order shown in S9A. Insertion of the IS1-family transposase in the gene encoding a hypothetical protein significantly reduces its expression in the mutant ($p<0.0001$), while the IS1-family transposase is transcribed instead (average TPM ~70 at 26° C and ~118 at 19° C). Consequently, this insertion causes a significant decrease in expression/little activity ($p <0.001$) of an ISL3-family transposase downstream of the hypothetical protein in Δ*mcyB* compared to the PCC 7806 wildtype.

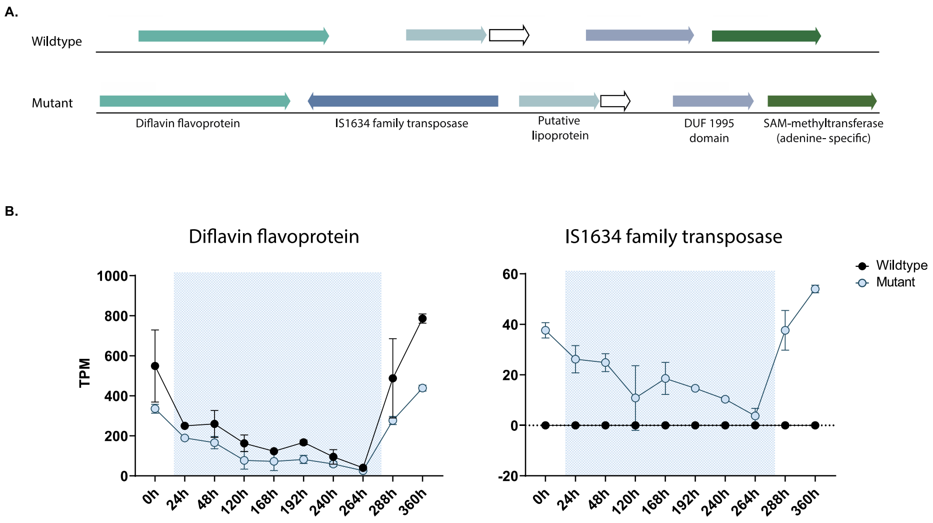

Figure S10 (A): In *Microcystis aeruginosa* PCC_7806 Δ*mcyB*, an IS1634 transposase inserted between a diflavin flavoprotein and a putative lipoprotein. B) The IS1634 transposase inserted in the Δ*mcyB* strain mimics gene expression of the diflavin flavoprotein.

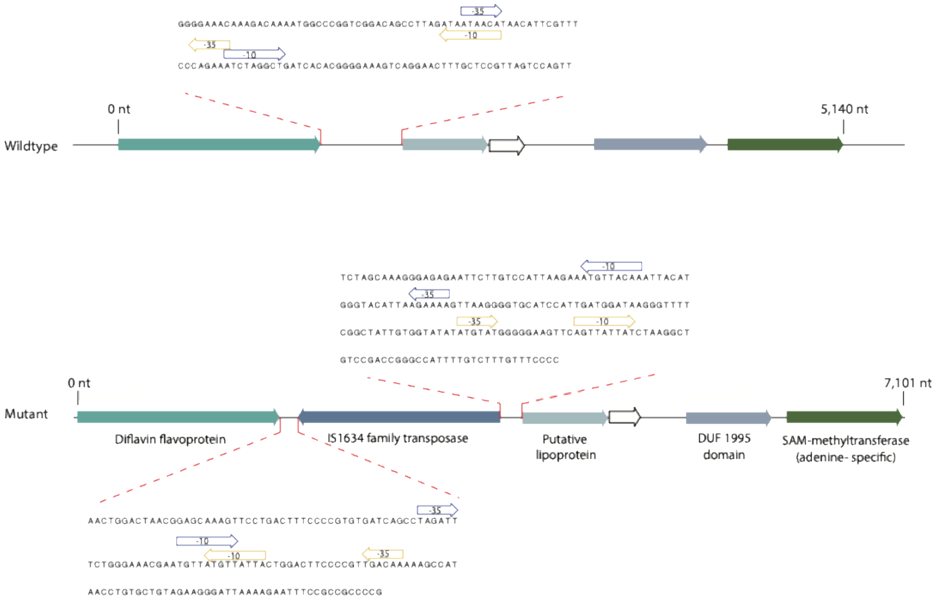

Figure S11: Predicted promoter regions found by BPROM [2]. The predicted promoter regions varied between the PCC 7806 wildtype and Δ*mcyB*. Insertion of the IS1634 family transposase in- Δ*mcyB* created new putative promoter regions, before the putative lipoprotein and the diflavin flavoprotein. Putative promoters are colored based on whether they are on the positive strand (blue), versus negative strand (yellow).

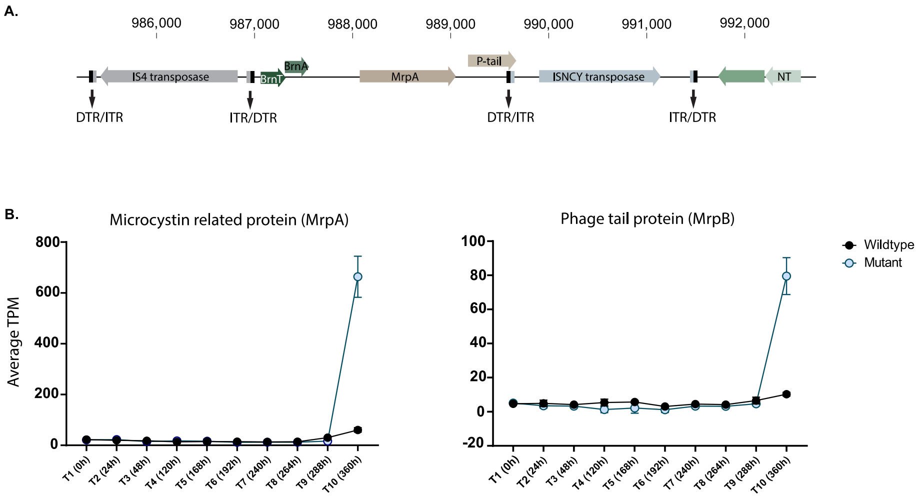

Figure S12: A gene encoding a phage tail protein, P-tail (previously annotated as *mrpB*) was found in *Microcystis aeruginosa* PCC7806, next to a microcystin-reliant protein (*mrpA*). These two genes were situated near a BrnTA toxin-antitoxin system (which is flanked by an IS4-transpoase), and an ISNCY (insertion element not characterized yet). B) Normalized gene expression (average TPM), for *mrpA* and the phage tail gene. These two genes appear to be active in the mutant at one time point, potentially providing some sort of function to the cells.

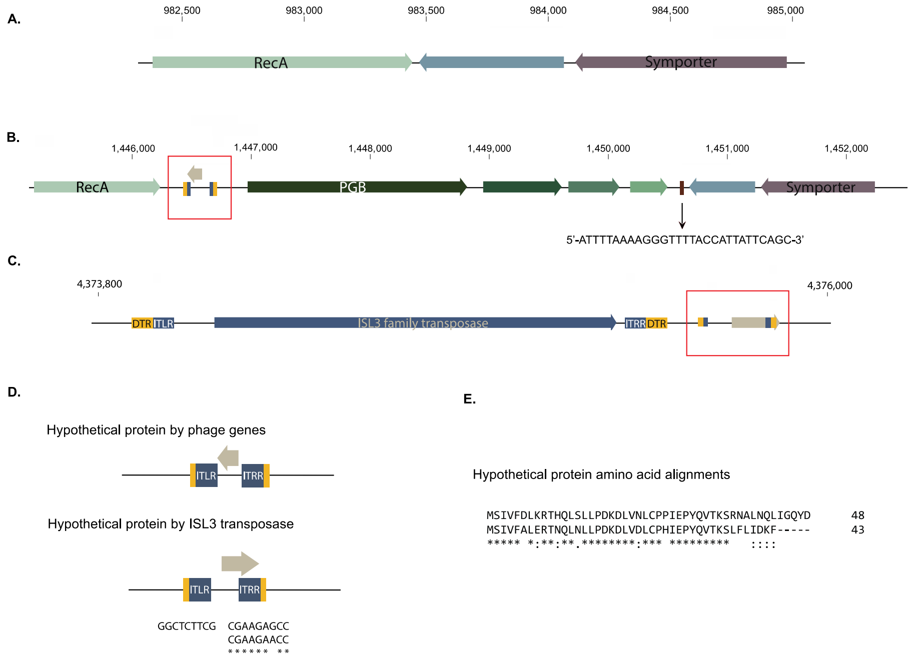

Figure S13: A phage protein gene with peptidoglycan binding domains (PGB- dark green) was identified in *Microcystis aeruginosa* NIES 843. Figure A is an unmutated gene arrangement in *Microcystis aeruginos*a LE3. Genes with similar colors are homologous to one another. B) In *Microcystis aeruginosa* NIES843, the phage gene (PGB) was found next to three other unique genes (nucleotide sequences not found in other complete *Microcystis* spp. genomes) downstream of PGB (all green) may have also been acquired with this gene. Flanking these four genes upstream is a gene encoding a hypothetical protein (marked by a red box) which appears to have been inserted by a ISL3 family transposase, as it has similar inverted terminal repeats to ISL3 and is flanked by direct terminal repeats, and a homolog of this gene is present in another area of the genome (seen in figure III, marked by a red box), next to an ISL3 transposase (II, IV, V). Downstream of these four genes is a sequence which is repetitive in the genome (exists in 25 areas). This sequence is 5'-ATTTTAAAAGGGTTTTACCATTATTCAGC-3'. Of the twenty-five repeats, ten are by transposases, and of those ten, four are by TnpB transposable elements. Similar gene arrangements without the four acquired genes are found in other *Microcystis* genomes (*Microcystis aeruginosa* NIES-88, LE3 and *Microcystis virdis* NIES-102 were checked).

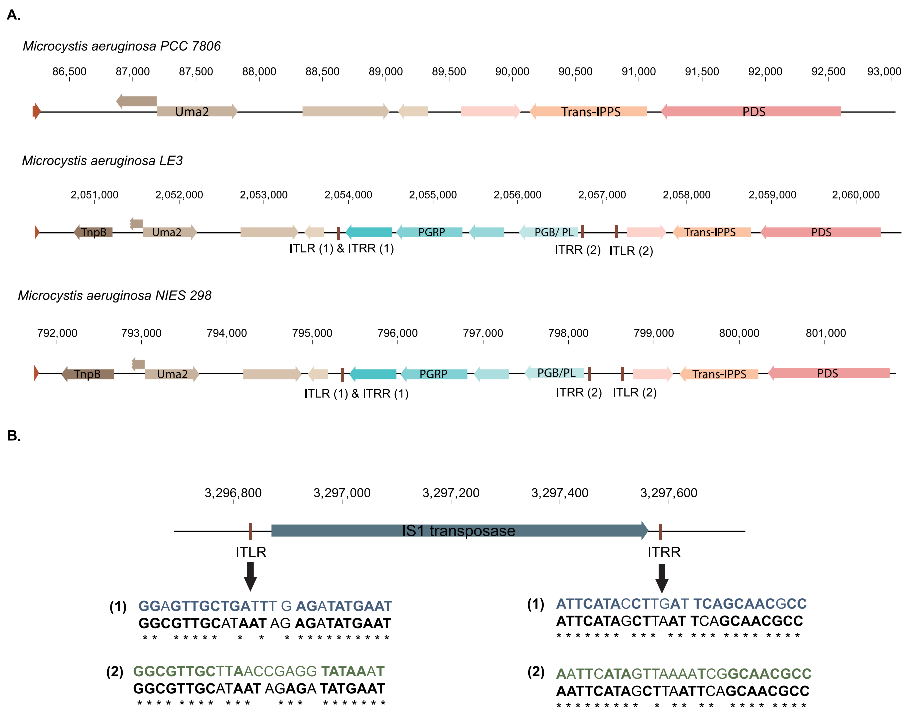

Fig S14: A) *Microcystis aeruginosa* LE3 and *Microcystis aeruginosa* NIES298 both have a putative phage tail lysozyme (PGB/PL gene). Along with this gene are three other genes (in blue) that may have been acquired with the putative phage (PBG/PL) gene, one of them has a peptidoglycan recognition/binding protein domain (PGRP). In *Microcystis aeruginosa* LE3 and *Microcystis aeruginosa* NIES298, these four genes are flanked by mutated areas which have homology IS1-family transposases in the genome. These intergenic regions have areas that align with the inverted terminal repeat regions of an IS1-family transposase (ITLR – inverted left terminal repeat, ITRR- inverted right terminal repeat). ITLR (1) is an imperfect ITR of ITRR (1). In other *Microcystis* strains (NIES843, PCC 7806), the gene arrangement is similar to LE3 and NIES298, where an uma2 endonuclease is located near a tRNA, and a phytoene synthase (Trans-IPPS) and a phytoene desaturase (PDS) are present. The unlabeled genes are uncharacterized genes.

B) Alignment of the inverted terminal repeats for an IS1-family transposase. The colored sequences are those from the intergenic regions in LE3 and NIES298, and black are the sequences of the IS1-transposase ITR's. The numbers by the sequences correspond to the location of the ITLR and ITRR, above.

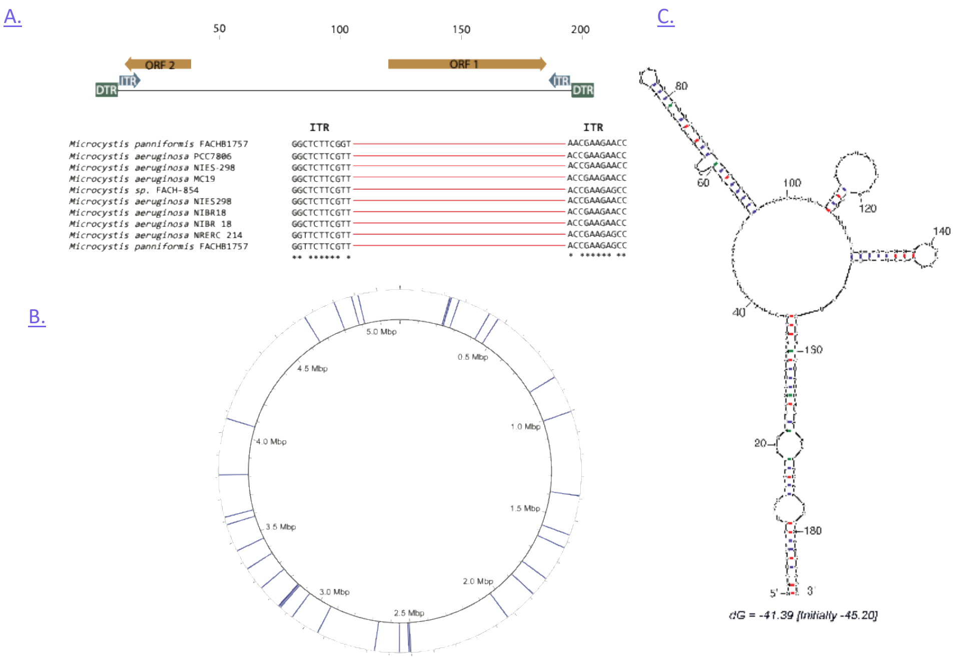

Fig S15: A) Simplified overview of the 187-nt insertion sequence in the *sbp* gene in Δ*mcyB*. The insertion sequence is flanked by 8-nt long direct terminal repeats, duplicated from the target site. There are also imperfect 11-nt long inverted terminal repeats (ITR), and two putative open reading frames. Homologous insertion sequences from other *Microcystis spp*. show similar ITR's. B) Locations of identical 187-nt MITEs in the Δ*mcyB* genome. C) *In-silico* Mfold [3] prediction of the RNA secondary structure of the 187-nt MITE sequence (Predicted ΔG= -41.39).

Formatted: Centered

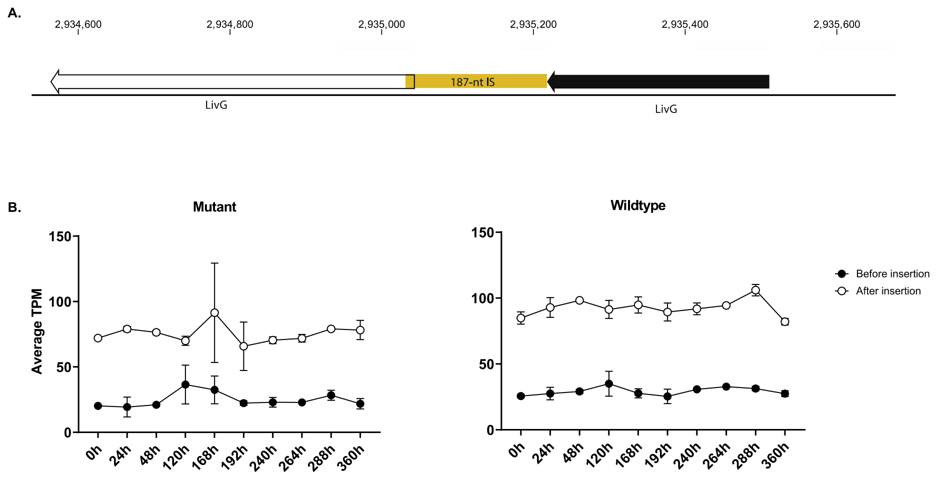

Fig S16: A) 187-nt long insertion sequence (IS) was inserted in a gene encoding a branched chain amino acid transporter (LivG) in the mutant strain. B) The *livG* gene shows expression values roughly half that of the wildtype, before and after the insertion sequence. This is unlike the sulfate binding protein in the mutant, which has the same 187-nt IS, but has upregulated expression of sulfate transporter gene cluster (*sbp-cysTWA*).

```
MITE              GGCTCTTCGTTACTTGTTATGGTTCGATAGGGGGGCATAAATCA----------------    44
ISL3 Transposase  GGCTCTTCGTTACTCTTTATGCTAAATCAACAGACAAGATGCCAAGATAACATACTTCTA    60
                  **************  ***** *     *   *   * *   **

MITE              ---------------------------------------------------------ACA    47
ISL3 Transposase  TCCTCTATCATTCTTTGAATCTCTTCTTCCGTTACGTCATTTCTTCGGCTAACATTTAAA    900
                                                                           * *

MITE              AAATCTTTATCTG-----------------------------------------------    60
ISL3 Transposase  ATATCTCCTTCTTTTAATTGTTCGAGTATATTCTCGGCTAGTCTTTTCGTATAGGTTCGT    960
                  * ****   ***

MITE              ----------------------------GTAAGGGATTTAATTGATTAGTTCGCTTTAGA    92
ISL3 Transposase  TTCTTGGCGACAAAATCTAACTCTTCACTAAAGGGTTTTCGACAATTATCGCACTT-AAA    1019
                                                ***** ***     ****   * *** * *

MITE              TAAAAAACAATTGACAA-------------------------------------------    109
ISL3 Transposase  TTGACGACGATTAACCTGTAGGTACACTGGTTGACCTGAGATTGGTAAATCTTTGACTAA    1079
                  *  *  ** *** **

MITE              -------------------------------------------AAATCGCCCAATGTCTT    126
ISL3 Transposase  ATGTCGATGATTTTGGTGGAGTTTATCGCTCTCTAACCCACAACGAGGACAGATTGCTTT    1139
                                                               *   *  * **  **

MITE              TCTCTATAAGGGTTCCATCC----------------------------------------    146
ISL3 Transposase  TTGATTTTTCGATTCGATTTGGCAAACTATACCGATATTTTCTAGGTGTAGATAGCCTTG    1199
                  *   * *   * *** **

MITE              ------------------------------------------------------------    146
ISL3 Transposase  AATAGACGTTCCTTTTAGGTTCAAAAATTTGTCAAGTATCATAAGTAAAATACTCTGGTT    1259

MITE              -------------------CTTATAACTCCCGT---------------------------    160
ISL3 Transposase  TTTGGCTATTATATCAAATCTTAACTCGATTGTCTATCTTTTGATATTAACTTGTTTGTG    1319
                                     ****   *    **

MITE              ------------------------------------------------------------    160
ISL3 Transposase  CCGTAAGTGTCTCCCTGTATAGATTTCAGCTACTTTTAGGCGTAGGTACTCTCATCGAAT    1379

MITE              ----------------CTATTGCATAAGACAAACCGAAGAACC    187
ISL3 Transposase  TTTATTTTAAGTTAATTATTTGCATAACAATTCCCGAAGAGCC    1422
                                     ******** *    ******* **
```

Fig S17: Shortened alignment (Clustal Omega output) of an ISL3-family transposase with inverted terminal repeats (ITR) that match the imperfect ITR’s of the 187-nt miniature inverted repeat transposable element that is active in *Microcystis aeruginosa* PCC 7806.

A.

```
Wildtype   MQSSQSYLSLLMATVLTGGMLASCTSSPTNSGNDSQKSVTVTLVSYAVTQSAYEKIIPKF    60
Mutant     MQSSQSYLSLLMATVLTGGMLASCTSSPTNSGNDSQKSVTVTLVSYAVTQSAYEKIIPKF    60
           ************************************************************

Wildtype   VEEWQQKTGQKVTFEQSYGGSGSQTRAVIDGLEADVVALALALDTKKIEQAGLIQPGWEK    120
Mutant     VEEWQQKTGQKVTFEQSYGGSGSQTRAVIDGLEADVVALALALDTKKIEQAGLIQPGWEK    120
           ************************************************************

Wildtype   EAPNDSIVHKSVVAFVPRDANIKINKWSDLAKDNIKVITANPKTSGGARWNFLALWGSVT    180
Mutant     EAPNDSIVHKSVVAFVPRDANIKINKWSDLAKDNIKVITANPKTSGGARWNFLALWGSVT    180
           ************************************************************

Wildtype   QAGGSEQEAQTFVEKVFKNAPVLPRDARESSDVFFKQGQGNVLINYENEMILANQKGEKL    240
Mutant     QAGGSEQEAQTFVEKVFKNAPVLPRDARESSDVFFKQGQGNVLINYENEMILANQKGEKL    240
           ************************************************************

Wildtype   AYTVPTDYNISIDNPVAVVDANVDKKGTRKVAEAFVQFLFTPEAQREFAKVGFRPVEPTI    300
Mutant     AYTVPTDYNISIDNPVAVVDANVDKKGTRKVAEAFVQFLFTPEAQR--------------    286
           **********************************************

Wildtype   VKEFESKFPQIKNLFTVQDLGGWDKVQKQFFDDGGLFDKILTKAGKS    347
Mutant     -----------------------------------------------    286
```

B.

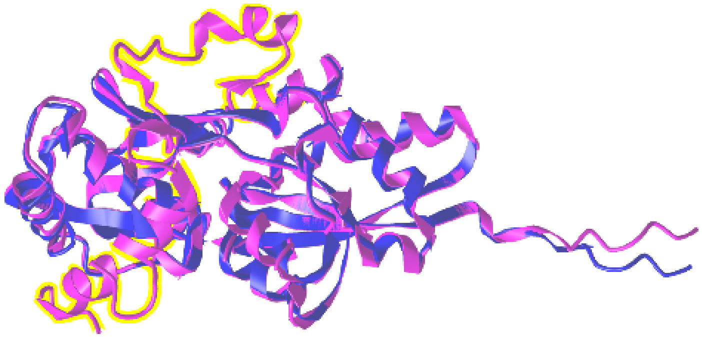

TM-align score (normalized by mutated sbp) : 0.98614
TM-align score (normalized by wildtype sbp): 0.80152
RMSD: 0.79
Aligned length: 262

Figure S18: A) Clustal Omega alignments [4] of the wildtype and Δ*mcyB* translated *sbp* gene products. Outlined in red are the conserved amino acid residues involved in sulfate binding, as identified by NCBI conserved domain search [5, 6]. B) Protein structure alignments of the two *sbp* translated gene products. The pink structure is the wildtype *sbp* gene product, and blue is the Δ*mcyB* gene product. The highlighted yellow region is the area of the wildtype protein that is missing in the coding region before the MITE insertion in Δ*mcyB*.

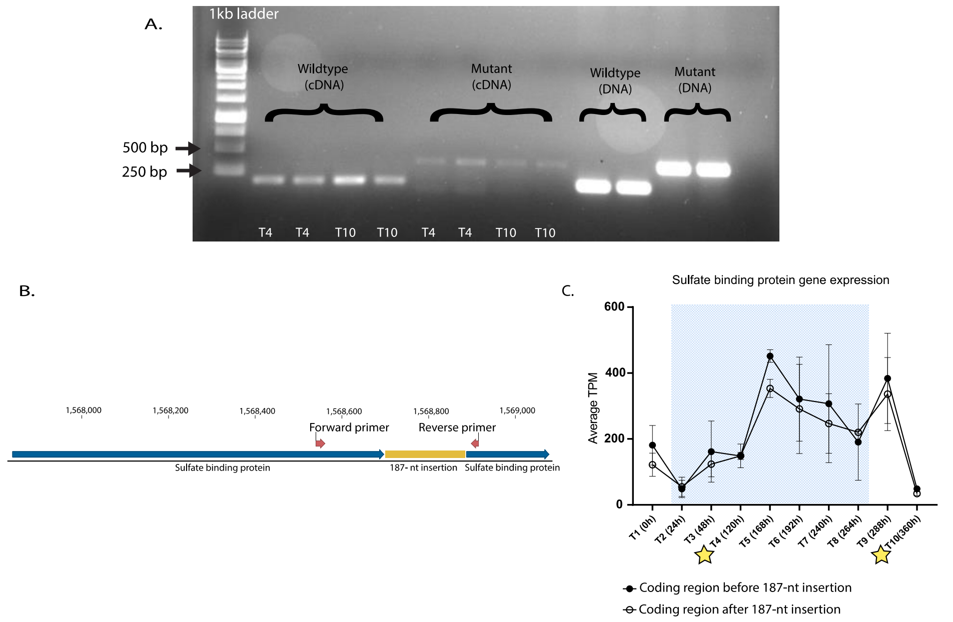

Fig S19: A) Results from reverse transcriptase PCR on different timepoints taken from the original RNA-seq experiment [1] .Transcriptomic libraries from the original experiment were used in this manuscript. In the *Microcystis aeruginosa* PCC 7806 wildtype, a 181-bp amplicon was generated, consistent with the lack of the MITE in the gene encoding a sulfate binding protein. For the PCC 7806 Δ*mcyB* strain, faint bands around 376-bp in length were generated, consistent with the presence of the MITE insertion in the gene encoding the sulfate binding protein. B) Points that RT-PCR analysis was done are indicated with a star in the RNA-seq time series. This gel image has not been modified beyond the text added to the figure. All bands present were run on the same gel at the same time.

Supplemental References

1. Stark GF, Martin RM, Smith LE, Wei B, Hellweger FL, Bullerjahn GS, McKay RML, Boyer GL, Wilhelm SW: **Microcystin aids in cold temperature acclimation: Differences between a toxic *Microcystis* wildtype and non-toxic mutant**. *Harmful Algae* 2023, **129**:102531.

2. Solovyev VV, Salamov A: **Automatic annotation of microbial genomes and metagenomic sequences**. In: *In Metagenomics and its Applications in Agriculture, Biomedicine and Environmental Sciences.* Edited by Li RW: Nova Science Publishers; 2011: 61-78.

3. Zuker M: **Mfold web server for nucleic acid folding and hybridization prediction**. *Nucleic Acids Res* 2003, **31**(13):3406-3415.

4. Madeira F, Pearce M, Tivey ARN, Basutkar P, Lee J, Edbali O, Madhusoodanan N, Kolesnikov A, Lopez R: **Search and sequence analysis tools services from EMBL-EBI in 2022**. *Nucleic Acids Res* 2022, **50**(W1):W276-W279.

5. Marchler-Bauer A, Bo Y, Han L, He J, Lanczycki CJ, Lu S, Chitsaz F, Derbyshire MK, Geer RC, Gonzales NR *et al*: **CDD/SPARCLE: functional classification of proteins via subfamily domain architectures**. *Nucleic Acids Res* 2017, **45**(D1):D200-D203.

6. Wang J, Chitsaz F, Derbyshire MK, Gonzales NR, Gwadz M, Lu S, Gabriele, James, Thanki N, Roxanne *et al*: **The conserved domain database in 2023**. *Nucleic Acids Res* 2023, **51**(D1):D384-D388.
